# A review of soluble mediator and cytokine networks linking combination immunotherapy, tumor microenvironment remodeling and skeletal muscle dysfunction

**DOI:** 10.3389/fimmu.2026.1883135

**Published:** 2026-08-04

**Authors:** Jiaxuan Wu, Ying Lu, Jiaqi Han, Bing Mu, Xinwei Wu, Xiaolin Wu, Yongkui Yu, Huixian Li, Shuang Ma

**Affiliations:** 1 School of Information Science and Engineering, Shenyang Ligong University, Shenyang, China; 2 School of Traditional Chinese Medicine, Beijing University of Chinese Medicine, Beijing, China; 3 Department of Anesthesiology, National Cancer Center, Chinese Academy of Medical Sciences and Peking Union Medical College, Beijing, China; 4 National Clinical Research Center for Cancer, Chinese Academy of Medical Sciences and Peking Union Medical College, Beijing, China; 5 Cancer Hospital, Chinese Academy of Medical Sciences and Peking Union Medical College, Beijing, China; 6 School of Mathematics and Statistics, Liaoning University, Shenyang, China; 7 Departments of Thoracic Surgery, The Affiliated Cancer Hospital of Zhengzhou University, Zhengzhou, China

**Keywords:** cancer cachexia, combination immunotherapy, cytokine network, skeletal muscle dysfunction, tumor microenvironment

## Abstract

Combinatorial immunotherapies have revolutionized the clinical management of advanced malignancies, yet their prominent anti-tumor efficacy is frequently compromised by treatment-induced skeletal muscle dysfunction governed by reciprocal signaling circuitry bridging the tumor microenvironment (TME) and peripheral muscle compartments. This review systematically delineates conserved soluble mediator and cytokine networks underlying therapy-triggered myotoxicity across five core combinatorial regimens, chemoimmunotherapy, targeted immunotherapy, gene therapy, tumor vaccines, and CAR-T cell therapy, alongside modality-specific toxic signaling axes. Cancer-associated fibroblast-derived TGF-β, glycolysis-originated lactate, and IL-6/TNF-α-centered inflammatory cascades converge to activate the myostatin/FoxO3 transcriptional program and ubiquitin-proteasome proteolysis, disrupting muscle anabolic-catabolic homeostasis. Type II fast-twitch glycolytic fibers and muscle satellite cells display disproportionate susceptibility to inflammatory and metabolic damage, whereas clinical confounders including corticosteroid exposure, chronological age, and sex hormones stratify interpatient vulnerability to muscle wasting. Conventional and emerging skeletal muscle biomechanical assessment modalities are critically benchmarked, with electrical impedance myography highlighted as a high-sensitivity platform for subclinical tissue remodeling detection, and tiered multimodal monitoring pipelines are formulated for prospective immunotherapy clinical trials. Translational muscle-protective interventions covering structured resistance training, personalized nutritional supplementation, and anti-cachexia pharmacotherapy are comprehensively consolidated. Collectively, this review establishes an integrated mechanistic and translational framework to advance holistic oncologic care that reconciles robust anti-tumor immunity with sustained skeletal muscle integrity.

## 1 Introduction

Cancer is a global disease that significantly threatens human health owing to its high incidence and mortality, posing substantial public health concerns. While traditional chemotherapy plays a crucial role in treating metastatic cancer, its systemic toxicity, notably its potential for skeletal muscle dysfunction, often leads to a decline in patient quality of life and reduced treatment tolerance. The advent of cancer immunotherapy, especially targeting immune checkpoint inhibitors, has shown remarkable efficacy in activating the immune system. However, monotherapy efficacy is restricted owing to the immunosuppressive characteristics of the tumor microenvironment (TME), leading to the exploration of combination strategies, such as chemotherapy and targeted therapy, as key directions to enhance efficacy (1). Notably, the impact of these combined treatments on skeletal muscle biomechanics and the underlying mechanisms remains unclear (2). Given the pivotal role of skeletal muscle in movement and metabolism, alterations in its structure and function can not only impair patient mobility, but may also interact with the TME to influence treatment outcomes. Therefore, investigating the interplay between skeletal muscle biomechanics, the TME, and immunotherapy is of significant scientific and clinical importance (3).

Skeletal muscle constitutes 40%-50% of the body’s mass, serving not only as a core component of the musculoskeletal system but also plays a vital role in maintaining body homeostasis through energy metabolism, hormone regulation, and other physiological processes (4). Regarding cancer treatment, the skeletal muscle is susceptible to the toxic effects of chemotherapy drugs, inflammation induced by immunotherapy, and multiple pleiotropic effects of cytokines in the TME (such as IL-6 and TNF-α). These factors can precipitate biomechanical abnormalities such as muscle atrophy, strength reduction, and changes in stiffness, which in turn contribute to the development of cachexia and reduce treatment adherence, creating a vicious cycle of “tumor-muscle” deterioration (5). Therefore, evaluating the dynamic alterations in skeletal muscle biomechanics during cancer treatment and analyzing their interaction with the TME and immunotherapy are essential steps to optimize treatment plans and improve patient prognosis.

The TME plays a crucial role in immune evasion and tumor progression, creating a complex network that resists immune attack through the infiltration of immunosuppressive cells, accumulation of inhibitory cytokines, and remodeling of matrix fibrosis. Recent studies have suggested that there is bidirectional regulation between skeletal muscle and the TME, and inflammatory mediators and metabolic byproducts within the microenvironment (such as TGF-β and lactate) can induce muscle cell apoptosis and protein breakdown via the bloodstream, while myokines (such as irisin) may regulate tumor cell metabolism and influence immunotherapy responses (6). However, a comprehensive understanding of how immune combination therapies mediate muscle damage through the TME and how skeletal muscle dysfunction affects treatment outcomes remains elusive (7).

Therefore, this review was designed to systematically delineate the soluble mediator and cytokine networks through which five distinct combination immunotherapy regimens—chemotherapy, targeted therapy, gene therapy, tumor vaccines, and CAR-T cell therapy—drive skeletal muscle dysfunction via TME remodeling; to characterize the differential vulnerability of fast- and slow-twitch muscle fibers and the confounding contributions of corticosteroids, age, and sex; to critically appraise traditional and emerging biomechanical assessment modalities with respect to their early detection sensitivity and suitability for clinical trial integration; and to consolidate evidence-based muscle-protective intervention strategies, thereby providing a structured mechanistic and translational framework to guide clinical practice and future research.

## 2 Formation and role of the immune ecological niche in the tumor microenvironment

Previous reviews including our prior work (4) primarily summarized immune-related muscle adverse events induced by single immune checkpoint inhibitors. In contrast, this review systematically integrates immunotherapy combined with chemotherapy, targeted therapy, gene therapy, tumor vaccines, and CAR-T cell therapy, highlighting five distinct regimens’ differential skeletal muscle toxicity, heterogeneity of fast- and slow-twitch fiber susceptibility, confounding effects of corticosteroids, and muscle protective interventions encompassing nutrition and exercise. This structural distinction explicitly defines the innovation boundary of the present review, differentiating it from earlier publications.

### 2.1 Mechanisms of immune ecological niche formation and maintenance

**Figure 1:**
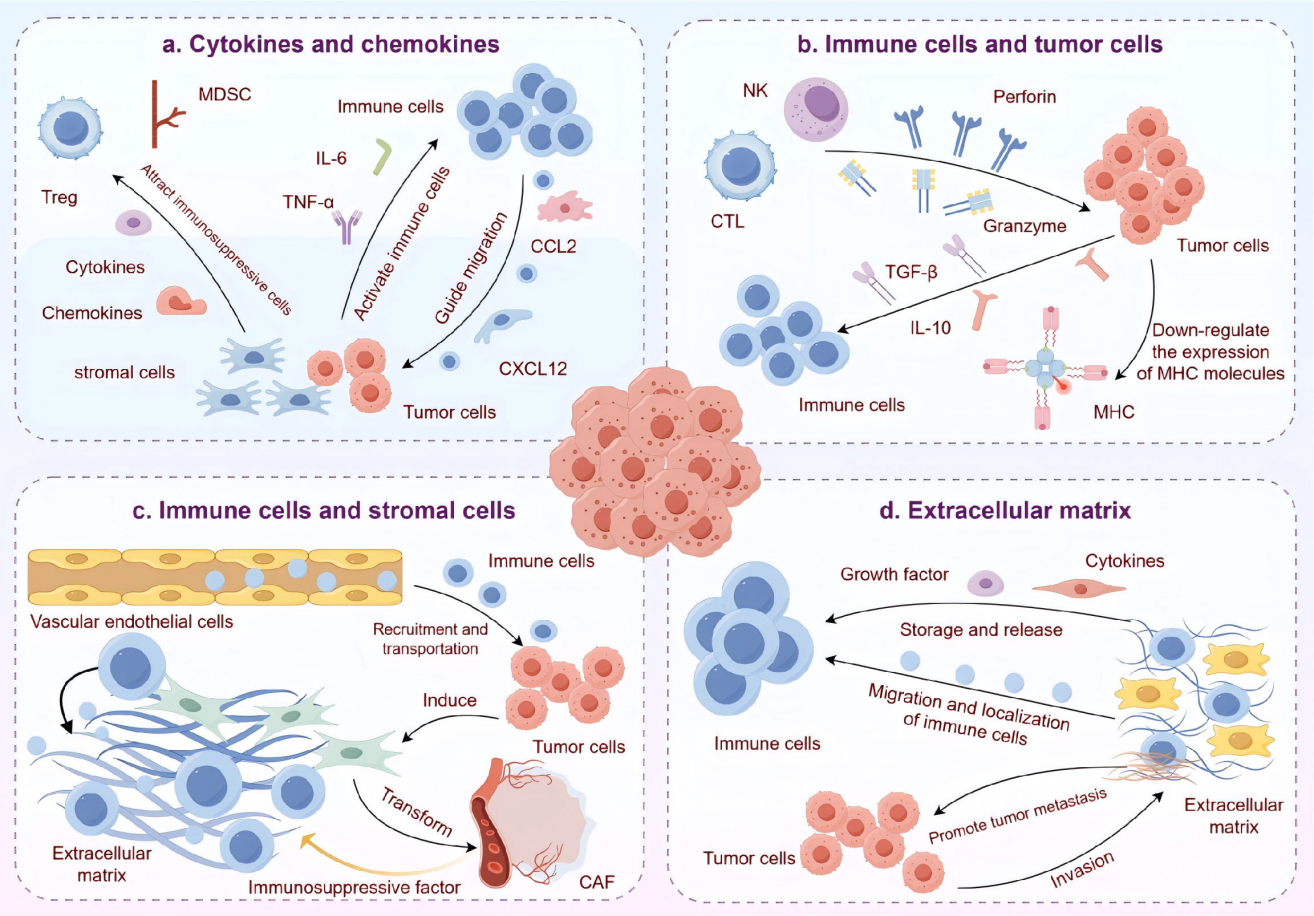
Dynamic balance of immune regulatory network and immune evasion mechanism in the TME. **(a)** Cytokines (including IL-6 and TNF-α) and chemokines (such as CCL2, CXCL12) secreted by tumor cells and stromal cells activate immune cells, guide migration, and also recruit immune-suppressive cells (Tregs, MDSCs); **(b)** Immune cells (NK, CTL) kill tumor cells through perforin and granzymes, while tumor cells secrete TGF-β, IL-10 to downregulate MHC molecules for immune evasion; **(c)** Endothelial cells participate in the transportation of immune cells, while CAFs produce immune-suppressive factors; **(d)** ECM stores and releases factors that affect immune cell migration and localization, promoting tumor metastasis and invasion.

Tumorigenesis and disease progression involve the interaction of multiple factors, not only related to the characteristics of tumor cells, but also closely associated with the TME (8). The TME is a dynamic system composed of heterogeneous components, including tumor cells, immune cells, stromal cells, and extracellular matrix (ECM), forming a unique ecological system. The concept of the immune ecological niche underscores the critical role of specific regions within the microenvironment in regulating immune cell functions (4). Within the TME, various cells secrete signaling molecules to form a complex regulatory network. Cytokines, including IL-6and TNF-α, released by tumor and stromal cells serve both as activating signals that enhance immune cell activity, and as chemoattractants that induce immune cell migration through specific concentration gradient (9) chemokines such as CCL2and CXCL12 bind to their corresponding receptors to form chemotactic pathways that regulate immune cell infiltration. Tumor cells also harness this mechanism by regulating the secretion of cytokines and chemokines to selectively attract immune-suppressive cells, including regulatory myeloid-derived suppressor cells (MDSCs) and T cells (Tregs), thereby constructing an immune microenvironment conducive to their own proliferation (**Figure 1a**) (10).

The immune system and tumors maintain a dynamic balance in their interactions. Effector immune cells, including natural killer (NK) cells and cytotoxic T lymphocytes (CTLs), can initiate cytotoxic programs via antigen recognition mechanisms and release perforin, granzymes, and other effector molecules. Meanwhile, tumor cells have evolved multiple immune evasion strategies, such as downregulating major histocompatibility complex (MHC) molecule expression to reduce antigen presentation efficiency and secretion of inhibitory cytokines, such as TGF-β and IL-10, to weaken immune responses. Furthermore, tumor cells can remodel stromal cells into cancer-associated fibroblasts (CAFs), which collectively create an immune-suppressive microenvironment (**Figure 1b**) (9).

The extracellular matrix network in the microenvironment exerts a double-edged effect. The ECM secreted by CAFs provides a physical scaffold that facilitates immune cell migration; however, its remodeling process may also lead to the release of sequestered growth factors that affect immune responses (11). While endothelial cells regulate immune cell infiltration through adhesion molecules, tumor-induced vascular abnormalities may hinder the infiltration of effector cells. Notably, stromal cells can transform into CAFs under the influence of the tumor, secreting suppressive factors that promote immune evasion (**Figure 1c**) (12). The biochemical properties of the ECM directly affect the formation of the immune ecological niche. Components, such as collagen and fibronectin, mediate immune cell localization via integrin receptors, and their degradation products can promote tumor metastasis and active immune responses (13). Dynamic remodeling of the ECM, coupled with the release of stored cytokines, constitutes a key aspect of microenvironmental regulation (**Figure 1d**) (14).

Comprehensive exploration of the formation mechanisms of the immune ecological niche requires the application of cutting-edge technologies. The immune synapse is a unique structure formed between immune cells and target cells that plays a critical role in the activation and cytotoxic functions of immune cells (15). In the TME, the formation and dynamic changes in the immune synapse are crucial for immune cell recognition and tumor cell killing (15). Through dynamic modeling, spatiotemporal changes in molecular interactions at the immune synapse can be directly visualized (16). Using computer simulation techniques, dynamic changes in immune cell and tumor cell surface molecules during immune synapse formation can be studied, as well as the influence of these changes on immune cell activation and cytotoxic functions (17).

Single-cell sequencing technology provides a potent tool for understanding the heterogeneity and interactions of cellular subpopulations in the TME (16). By sequencing individual cells from tumor tissues, the gene expression profiles of each cell can be analyzed, leading to the identification of different cell subpopulations. Within the immune ecological niche, single-cell sequencing technology can reveal the interaction networks between immune cell subpopulations, as well as between immune cells and tumor or stromal cells (18). Studies have shown that various functional T cell subpopulations exist in the TME, such as memory T cells, effector T cells, and exhausted T cells, which regulate each other through cytokines and surface molecules, collectively affecting the tumor’s immune response (17). Single-cell sequencing technology can also be used to identify new cell subpopulations and modes of intercellular interactions, providing essential clues for developing personalized tumor immunotherapy strategies (19).

Mechanical stress is a crucial physical factor in the TME that can influence the biological behavior of both tumor and immune cells (20). Research on the effects of mechanical stress on programmed death ligand 1 (PD-L1) expression has attracted considerable attention over the past few years. As an important physical factor in the TME, mechanical stress can regulate PD-L1 expression on tumor cell surfaces. When tumor tissue is subjected to mechanical compression or stretching, intracellular signaling pathways are activated, leading to increased transcription and expression of PD-L1 (21). PD-L1 is an important immune checkpoint molecule that can bind to programmed cell death receptor 1 (PD-1) on immune cell surfaces, inhibiting the activity and function of immune cells, thereby promoting tumor immune evasion (22). This research direction opens new possibilities for tumor immunotherapy.

### 2.2 Immune ecological niche impact on tumor immunotherapy

The immune ecological niche within the TME critically influences immune effector cell function and therapeutic responses. Pro-tumor niches are characterized by accumulation of immunosuppressive molecules (TGF-β, IL-10), hypoxia, and aberrant ECM remodeling that collectively downregulate cytotoxic T lymphocyte (CTL) proliferation and activity (23–25).

For example, CAF-secreted CXCL12 restricts CD8+ T cell infiltration, while recruitment and expansion of MDSCs suppress anti-tumor immunity via arginase-1 driven L-arginine depletion and impaired integrin-mediated CTL migration (26).

### 2.3 Immune-muscle interaction pathways in the TME

Immune and skeletal muscle cells engage in complex crosstalk whose dysfunction contributes to immunotherapy-associated muscle toxicity (24).

#### 2.3.1 Neuroendocrine axis-mediated regulation

The neuroendocrine axis, including sympathetic, parasympathetic, and hypothalamic-pituitary-adrenal (HPA) systems, exerts systemic regulation over immune, muscle, and stromal cells through neurotransmitter and hormone release (25).

Sympathetic norepinephrine activation of β2-adrenergic receptors induces IFN-γ release from CD8+ T cells and activates skeletal muscle ubiquitin-proteasome degradation pathways promoting muscle atrophy (24). Conversely, parasympathetic α7 nicotinic acetylcholine receptor signaling suppresses NF-κB nuclear translocation, reducing macrophage IL-6 production but paradoxically promoting muscle satellite cell apoptosis associated with cachexia progression (27, 28). Cortisol oscillations within the HPA axis inhibit muscle protein synthesis and modulate CAF remodeling capacity impacting immune evasion and tumor progression (28, 29).

#### 2.3.2 Cytokine network cross-tissue cascades

The cytokine milieu integrates immune-muscle dynamics and tumor progression. Tumor, stromal, and muscle cells secrete cytokines forming a network amplifying inflammation and modulating myogenesis.

IL-6 secreted by tumor and skeletal muscle cells establishes a JAK/STAT3 positive feedback loop that simultaneously promotes Th17 differentiation and suppresses myoblast differentiation, effects exacerbated by CAF-derived IL-6 (30–32). CAFs also secrete TGF-β and CCL2 which inhibit NK cytotoxicity and induce muscle atrophy (33).

TNF-α exerts a dual effect through the TNFR1/NF-κB pathway: it promotes dendritic cell maturation within the TME, while triggering serine phosphorylation of IRS-1 in skeletal muscle and disrupting insulin-dependent protein synthesis (34). CAF-derived IL-6 amplifies this inflammatory feedback loop and persistently activates STAT3 in muscle fibers. Co-activation of STAT3 and NF-κB together initiates the ubiquitin-proteasome protein degradation cascade, breaking the balance between muscle anabolism and catabolism (35). IFN-γ, while upregulating tumor antigen presentation via IRF1, may induce sustained demethylation of the muscle PD-L1 promoter, fostering localized immune suppression influenced by CAF-secreted IL-8 (36, 37).

The above inflammatory axes constitute only part of the muscle atrophy regulatory network. Multiple core signaling pathways closely associated with cancer cachexia and irAEs coordinate with STAT3/NF-κB and the ubiquitin-proteasome system to drive fiber loss. The myostatin/activin pathway acts as a master upstream suppressor of myogenesis: elevated myostatin activates FoxO3 transcription factors, which directly upregulate two key atrophy executors MuRF1 and Atrogin-1. In addition, impaired autophagy (mitophagy) leads to accumulated intracellular reactive oxygen species, further reinforcing inflammatory signaling and accelerating muscle catabolism. Collectively, myostatin/FoxO3, mitophagy and MuRF1/Atrogin-1 form a complete regulatory circuit coupled with cytokine-induced STAT3/NF-κB and ubiquitin-proteasome activation, jointly mediating therapy-triggered skeletal muscle dysfunction (32).

#### 2.3.3 Metabolic reprogramming synergy and antagonism

**Figure 2:**
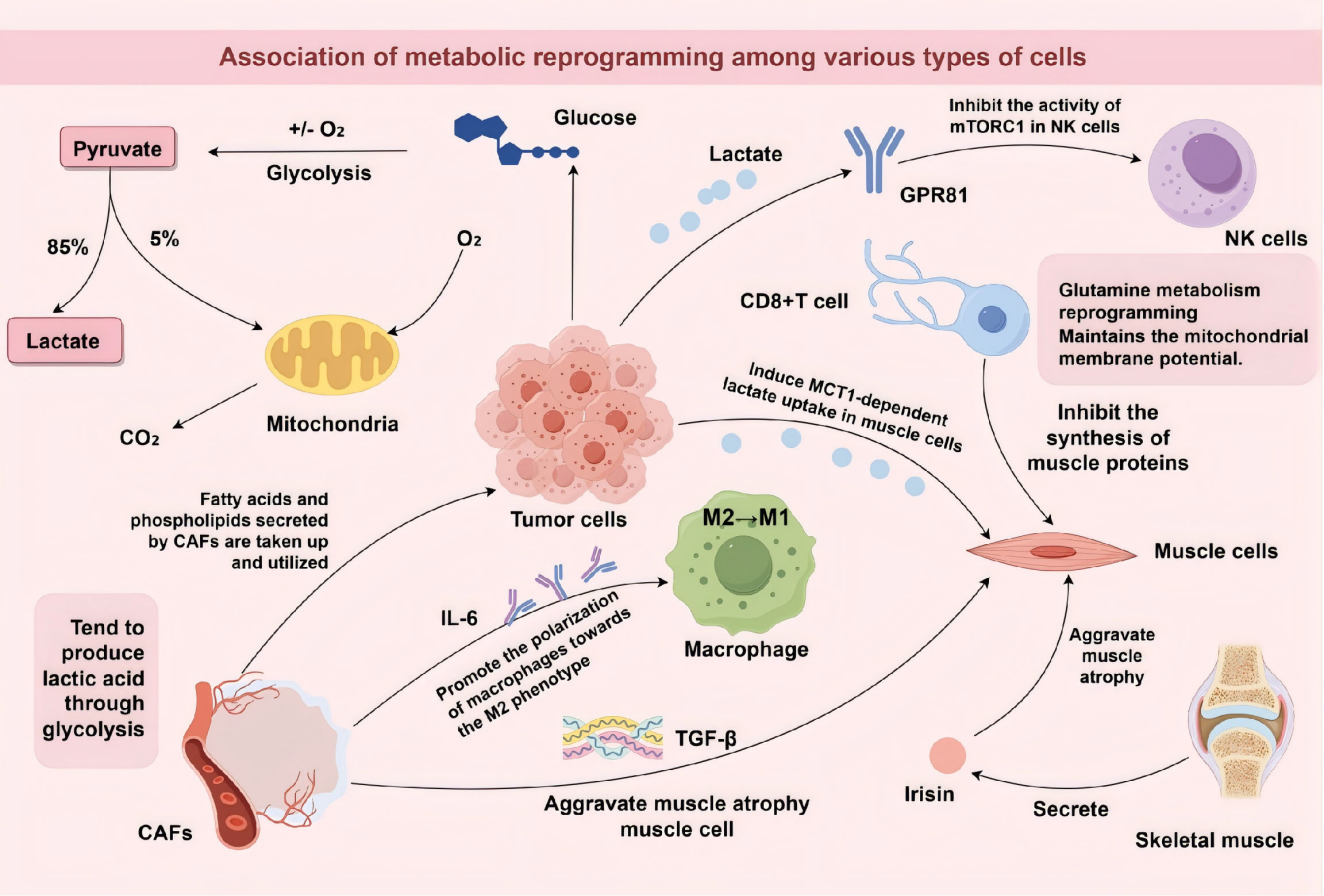
Metabolic reprogramming interactions between different cell types in the TME. Tumor cells produce lactate through glycolysis, a process known as the Warburg effect. Lactate inhibits mTORC1 activity in NK cells via GPR81 and induces MCT1-dependent lactate uptake in muscle cells. Cancer-associated CAFs tend to produce lactate through glycolysis and secrete fatty acids and phospholipids for uptake and utilization by tumor cells. CAF-secreted IL-6 promotes macrophage polarization to the M2 phenotype, whereas TGF-β exacerbates muscle atrophy. CD8+ T cells undergo glutamine metabolic reprogramming. Macrophages can transition from the M2 to the M1 phenotype. Muscle cells undergo metabolic changes, with irisin secreted by the skeletal muscles contributing to the worsening of muscle atrophy.

Within the TME, tumor, immune, and muscle cells undergo metabolic reprogramming, profoundly affecting tumor development, immune responses, and muscle function (38). CAFs play a key bridging role in metabolic reprogramming (**Figure 2**) (39). Tumor cells sustain their growth and proliferation through metabolic reprogramming, with the Warburg effect being a typical example. Lactate produced by this process elicits a series of effects (40). On one hand, it inhibits NK cell mTORC1 activity through the GPR81 receptor, weakening NK cell function. On the other hand, it induces muscle cell MCT1-dependent lactate uptake, thereby inhibiting mitochondrial oxidative phosphorylation, damaging the energy supply of muscle cells, and affecting muscle function (41). A portion of this lactate originates from the metabolism of CAFs, which tend to undergo glycolysis and produce lactate within the TME. In addition, tumor cells enhance glycolysis and alter fatty acid metabolism to obtain more energy and biosynthetic precursors. During this process, fatty acids and phospholipids secreted by CAFs are absorbed by tumor cells, supporting tumor cell growth and metastasis (42). Abnormal lipid metabolism in CAFs also affects immune cell function by secreting macrophage migration inhibitory factor (MIF) and creating an immune-suppressive microenvironment (43). In terms of amino acid metabolism, CAFs regulate the metabolism of glutamine and other amino acids to provide nitrogen sources for tumor cells, thereby supporting tumor cell growth. Glutamine metabolism also affects immune cell function by promoting Treg differentiation and suppressing the host immune response (43). Metabolic reprogramming within the TME has also been found to be significant. For example, macrophages can undergo metabolic reprogramming from an anti-inflammatory (M2) to a pro-inflammatory (M1) state, influencing the immune response of the tumor. CD8+ T cells undergo glutamine metabolic reprogramming, which helps maintain the mitochondrial membrane potential and supports T cell function (4). However, this process leads to competitive consumption of glutamine in the microenvironment, inhibiting muscle protein synthesis and negatively affecting the muscles (44). CAFs can alter the microenvironment of immune cells by secreting cytokines, thus influencing macrophage polarization and CD8+ T cell glutamine metabolism. In this respect, IL-6 secreted by CAFs may promote macrophage polarization to the M2 state, enhancing the immunosuppressive microenvironment of the tumor. Muscle cells in the TME also undergo metabolic changes. In addition to abnormal energy metabolism and reduced protein synthesis, skeletal muscle secretes the myokine irisin, which is involved in complex metabolic process (45). It mediates fat tissue lipolysis through PPARα, promotes tumor metastasis, and inhibits the expression of myogenesis regulatory factor Myostatin, forming a metabolic malignant cycle that further exacerbates muscle atrophy and other complications, severely affecting normal muscle function. CAFs can also influence muscle cell metabolism through paracrine effects, such as TGF-β secretion by CAFs, which may enhance the adverse effects of irisin on muscle cells, worsening muscle atrophy. Treatment strategies targeting CAFs may indirectly affect immune-muscle interaction pathways. For instance, by targeting the surface marker FAP on CAFs and using FAP-CART therapy to reduce CAF abundance, metastasis and tumor cell growth can be inhibited, immune cell function may be improved, and adverse effects on muscle cells may be alleviated. Inhibiting CAF activation, such as the use of TGF-β inhibitors, can reduce the secretion of cytokines by CAFs, regulate immune and muscle cell functions, and alleviate tumor-induced damage to muscle tissue (46). Modulating CAF metabolism and inhibiting metabolic synergy between tumor cells and CAFs may disrupt the malignant balance within the TME, positively affecting immune-muscle interaction pathways (42).

#### 2.3.4 Fast vs. slow twitch fiber heterogeneity and satellite cell dysfunction

Skeletal muscle fibers demonstrate heterogeneous vulnerability to immunotherapy-related damage. Fast-twitch glycolytic fibers (e.g., extensor digitorum longus, tibialis anterior) are more susceptible to cytokine-mediated atrophy and oxidative injury, whereas slow-twitch oxidative fibers (e.g., soleus) exhibit stronger anti-catabolic capacity due to greater mitochondrial antioxidant defenses and metabolic resilience (47).

Immune-related myotoxicity impairs satellite cell proliferation and differentiation critical for muscle regeneration. Sustained exposure to elevated TGF-β and TNF-α in the TME blocks satellite cell activation and self-repair functions by suppressing myogenic regulatory factors such as Pax7 and MyoD, shifting muscle damage from acute reversible injury to chronic wasting (32, 48).

In sum, these fiber-type differences and satellite cell impairments contribute to variation in muscle wasting severity and recovery potential among patients receiving combined immunotherapy.

## 3 The impact of immunotherapy combined with various methods on skeletal muscle biomechanics and its relationship with the TME

Combination immunotherapy regimens combining immune checkpoint inhibitors (ICIs) with chemotherapy, targeted therapy, gene therapy, tumor vaccines or CAR-T cell treatment have emerged as central therapeutic approaches in clinical oncology. These regimens synergistically strengthen anti-tumor immunity yet trigger complex skeletal muscle dysfunction through overlapping shared signaling axes and treatment-exclusive injury pathways. Prior literature and our group’s earlier reviews (4) largely focus on muscle adverse effects induced by single-agent ICIs. This work further expands analytical dimensions by stratifying shared pan-regimen mechanisms against unique toxic pathways across five combinatorial strategies (37).

Regardless of the specific combination strategy employed, multiple conserved TME-originated signaling cascades jointly drive skeletal muscle damage during combined immunotherapy. First, the CAF-TGF-β/SMAD3 fibrotic signaling axis serves as a core shared mediator of muscle structural remodeling. Cancer-associated fibroblasts (CAFs) function as the dominant cellular source of TGF-β inside the TME. Once local TGF-β levels surpass physiological thresholds, this cytokine binds muscle membrane TβRI/TβRII receptors and triggers sequential SMAD2/SMAD3 phosphorylation, resulting in excessive extracellular matrix deposition, elevated tissue stiffness and compromised muscle contraction-relaxation performance. Meanwhile, TGF-β stimulates tumor cell PD-L1 transcription to strengthen tumor immune escape and blunt the therapeutic response to ICIs (11). Second, lactate accumulation from Warburg-type glycolysis creates systemic metabolic disturbance harmful to both immune cells and muscle tissue. Tumor cells and CAFs generate massive lactate through aerobic glycolysis. Extracellular lactate binds NK cell-surface GPR81 receptors to suppress mTORC1-dependent cytotoxicity; simultaneously, lactate enters muscle cells via MCT1 transporters to block mitochondrial oxidative phosphorylation, curtail ATP synthesis, and disrupt intracellular pH as well as ion homeostasis critical for muscle function (49). Third, coordinated recruitment of myeloid-derived suppressor cells (MDSCs) and regulatory T cells (Tregs) establishes a local immunosuppressive and muscle catabolic microenvironment under all combination regimens. Combined treatment-induced inflammatory signals and immunogenic cell death broadly recruit MDSCs and Tregs, which secrete high levels of IL-10 and other anti-inflammatory cytokines to dampen effector T cell activity. MDSC-expressed arginase-1 continuously consumes tissue L-arginine, an indispensable precursor for skeletal muscle protein synthesis, thereby intrinsically impairing muscle anabolic capacity. Fourth, the interconnected IL-6/TNF-α inflammatory cascade acts as a central driver of muscle protein breakdown. Tumor cells, stromal fibroblasts and muscle fibers co-secrete IL-6 to build a self-amplifying JAK/STAT3 feedback loop. TNF-α exhibits dual tissue-specific effects mediated by TNFR1/NF-κB signaling: it facilitates dendritic cell maturation within the TME while triggering IRS-1 serine phosphorylation in muscle cells to disrupt insulin-mediated protein anabolism. CAF-derived IL-6 further amplifies this inflammatory loop and sustains persistent STAT3 activation in muscle fibers. Concurrent STAT3 and NF-κB activation collaboratively initiates the ubiquitin-proteasome degradation program and disrupts the intrinsic balance between muscle protein synthesis and breakdown. The above inflammatory signaling axes represent only a subset of the full muscle atrophy regulatory network. Multiple core cascades closely linked to cancer cachexia and immune-related adverse events (irAEs) operate in concert with STAT3/NF-κB and ubiquitin-proteasome signaling to induce myofiber loss (32). The myostatin/activin axis functions as a master upstream suppressor of myogenic differentiation: elevated myostatin triggers activation of FoxO3 transcriptional factors, which directly upregulate two key atrophy effector proteins MuRF1 and Atrogin-1. These two E3 ubiquitin ligases label contractile proteins such as actin and myosin heavy chain for proteasomal degradation, acting as the terminal executors of therapy-evoked muscle wasting. Besides transcriptional atrophy signaling, defective autophagy—especially impaired mitophagy, causes massive intracellular reactive oxygen species (ROS) accumulation, which further reinforces inflammatory transduction and accelerates muscle catabolism. Taken together, the myostatin/FoxO3 regulatory module, defective mitophagy and MuRF1/Atrogin-mediated protein degradation form an integrated circuit that cooperates with cytokine-triggered STAT3/NF-κB cascades to induce skeletal muscle dysfunction across all immunotherapy combination formats (32, 50). Fifth, PD-1/PD-L1 axis blockade leads to ubiquitous T cell-mediated inflammatory myopathy risk for any regimen containing ICIs. Disruption of PD-1/PD-L1 inhibitory signaling unleashes massive CD8+ T cell activation, which may facilitate aberrant T cell infiltration into skeletal tissue, accompanied by elevated serum creatine kinase and clinical manifestations of immune myositis (51).

Collectively, these overlapping TME-muscle crosstalk signaling networks lay the universal molecular framework underlying skeletal muscle injury triggered by all types of combined immunotherapy. Detailed toxic phenotypes unique to each combinatorial regimen are elaborated in the following subsections.

### 3.1 Immunotherapy combined with chemotherapy

The integration of chemotherapy and immunotherapy represents a notable breakthrough in the field of cancer treatment over the past few years (52). This combination therapy brings significant clinical benefits to patients through synergistic multipathway effects. The treatment plans, their effects, and their impacts are detailed in **Table 1** (52). As shown in **Table 1**, different PD-1 inhibitors exhibit distinct characteristics when combined with chemotherapy drugs (52). For instance, pembrolizumab is typically used in combination with cisplatin +5-FU or cisplatin/carboplatin for the treatment of various solid tumors, such as esophageal and cervical cancers (60). This combination therapy is based on a unique dual treatment mechanism. In terms of immune regulation, PD-1/PD-L1 inhibitors (such as pembrolizumab and nivolumab) block the immune evasion pathway of tumor cells, helping T cells restore their anti-tumor function (61). These drugs specifically bind to PD-1 receptors or PD-L1 ligands, effectively eliminating immunosuppressive signals in the TME and reactivating the immune system (62). Chemotherapeutic drugs also have a direct anti-tumor role. Cisplatin dysregulates genetic material replication by forming platinum-DNA adducts; cyclophosphamide, an alkylating agent, induces DNA crosslinking and strand breakage; albumin-bound paclitaxel improves drug delivery efficiency using nanocarrier technology, damaging the tumor cell cytoskeleton while reducing allergic reactions caused by traditional paclitaxel (63). For example, the combination treatment of esophageal cancer with pembrolizumab and cisplatin +5-FU not only directly kills tumors and releases antigens but also enhances subsequent immune responses by modulating the immune microenvironment (60). From the perspectives of both treatment efficacy and side effects, these combination therapies present both advantages and disadvantages (64). For instance, pembrolizumab combined with cisplatin +5-FU significantly prolongs survival, with better outcomes in patients with a PD-L1 CPS ≥ 10. However, it is also associated with adverse effects such as nausea, vomiting, and bone marrow suppression due to chemotherapy (53). Similar considerations apply to other combination therapies, such as nivolumab combined with oxaliplatin +5-FU/capecitabine, which demonstrates efficacy in Asian populations, although oxaliplatin may cause neurotoxicity. Different combination schemes exhibit varying effects on skeletal muscle (22). Cisplatin may induce bone pain and osteoporosis, whereas 5-FU can affect the nervous system, causing myositis and muscle pain. These effects typically vary depending on the drug combinations in different therapeutic schemes, and warrant close monitoring during treatment (53).

**Table 1:** FDA-approved PD-1 inhibitor combined chemotherapy regimens.

PD-1 inhibitor	Chemotherapy drug	Mechanism	Clinical efficacy and side effects	Applications in cancer	Effects on skeletal muscle	Risk-benefit balance & ASCO management recommendations	Refs.
Pembrolizumab	Cisplatin + 5-FU	PD-1 inhibitors block the binding of PD-1 and PD-L1, restoring immune cell activity; chemotherapy kills tumor cells, and the two work together to enhance the immune response.	Clinical Efficacy: Significantly prolongs survival, with better results in patients with PD-L1 CPS ≥ 10. Side Effects: Nausea, vomiting, bone marrow suppression, etc.	Used for various solid tumors, such as esophageal cancer, in combination with cisplatin and 5-FU.	Cisplatin may cause bone pain, osteoporosis, and long-term use may reduce bone density, increasing the risk of fractures. 5-FU affects the nervous system, causing myositis and muscle pain, especially in the soles of the feet. The combination of these two drugs, while killing tumor cells, may lead to a decrease in muscle strength and an increased risk of muscle atrophy through multiple pathways, such as affecting nerve, skeletal, and muscle metabolism.	Benefit: Meaningful OS improvement in PD-L1 CPS ≥ 10 disease. Risk: Overlapping platinum-fluoropyrimidine toxicity and ICI-mediated myositis may compound muscle injury. Management: Conduct baseline CK assessment and symptom review (consistent with ASCO baseline workup principles); monitor CK before each treatment cycle and serially during therapy; hold ICI and initiate corticosteroids for grade ≥ 2 immune-mediated myositis; permanently discontinue for myasthenia gravis-like symptoms or markedly elevated CK with severe muscle involvement (51).	(53)
Pembrolizumab	Cisplatin/Carboplatin	Blocks immune evasion pathways, chemotherapy activates the immune system and destroys tumor cells to attack the tumor.	Clinical Efficacy: Suitable for persistent/relapsed/metastatic cervical cancer with PD-L1 CPS ≥ 1. Side Effects: Possible nephrotoxicity (cisplatin-related), bone marrow suppression, etc.	For PD-L1 positive cervical cancer patients.	Cisplatin may cause bone pain and osteoporosis; carboplatin has no direct skeletal muscle toxicity reported, but may indirectly lead to muscle atrophy by affecting nutrient metabolism. Combined with immune modulation, it may exacerbate muscle damage.	Benefit: Clinically significant benefit in PD-L1-selected recurrent/metastatic cervical cancer. Risk: Platinum-related nephrotoxicity and myelosuppression may impair muscle nutrition; ICI adds immune myositis risk. Management: Baseline and interval CK assessment; ASCO-guided graded ICI interruption if myositis is suspected (51).	(54)
Nivolumab	Oxaliplatin + 5-FU/Capecitabine	PD-1 inhibitors remove immune suppression, chemotherapy kills tumor cells, and enhance immune cells’ recognition and killing of tumors.	Clinical Efficacy: Oxaliplatin combined with 5-FU is effective in Asian populations. Side Effects: Possible neurotoxicity (oxaliplatin-related).	Used for gastric cancer and other cancers, especially in Asian populations.	Oxaliplatin may cause neurotoxicity, affecting nerve control over muscles, leading to a decrease in muscle coordination. 5-FU can cause myositis and muscle pain. Capecitabine is converted into 5-FU in the body and can also lead to symptoms such as muscle pain. After combining with nivolumab, the immune system is activated, and while attacking tumor cells, the abnormal immune response may cause immune damage to skeletal muscles, further exacerbating muscle dysfunction.	Benefit: Improved outcomes in gastric cancer, particularly in Asian populations. Risk: Oxaliplatin neuropathy may impair neuromuscular control; fluoropyrimidine-related myalgia may compound immune-mediated muscle dysfunction. Management: Serial CK and neuromuscular symptom monitoring; suspend ICI per ASCO irAE algorithm for grade ≥ 2 immune-mediated myositis (51).	(55)
Tislelizumab	Cisplatin + Gemcitabine	Blocks the PD-1/PD-L1 signaling pathway, chemotherapy kills tumor cells, and synergistically enhances anti-tumor immunity.	Clinical Efficacy: Increases objective response rates. Side Effects: Includes hematologic toxicity and other chemotherapy-related side effects.	Used for the treatment of head and neck tumors and others.	Cisplatin can cause bone pain and osteoporosis. Patients treated with gemcitabine may experience flu-like symptoms, including muscle pain, and may also develop edema, with some patients experiencing localized pain. When used together, the adverse effects of cisplatin and gemcitabine on skeletal muscles are compounded. Additionally, during immune modulation by tislelizumab, changes in the immune microenvironment may affect the normal metabolism and repair of muscle tissue, increasing the risk of muscle pathology.	Benefit: Improved objective response rates in head and neck tumors. Risk: Hematologic toxicity narrows the therapeutic window; combined platinum-gemcitabine myotoxicity is escalated by immune activation. Management: Frequent CK and muscle symptom surveillance; apply ASCO irAE management pathway for suspected immune myopathy (51).	(56)
Sintilimab	Paclitaxel + Carboplatin	PD-1 inhibitors restore immune cell function, chemotherapy induces tumor cell apoptosis, and together they enhance immune surveillance and killing.	Clinical Efficacy: Demonstrates efficacy in the treatment of lung cancer and others. Side Effects: Paclitaxel may cause allergic reactions, neurotoxicity, and other side effects.	Used for solid tumors	Paclitaxel can affect peripheral nerves, leading to numbness and abnormal sensations in the hands and feet, and impairing normal muscle contraction and relaxation. Carboplatin may indirectly affect muscle health. When combined with sintilimab, immune modulation may disrupt the body’s original balance, altering muscle sensitivity to chemotherapy drugs and exacerbating muscle weakness, atrophy, and other related issues.	Benefit: Efficacy demonstrated in resectable and advanced lung cancer. Risk: Taxane peripheral neuropathy impairs muscle contraction; immune modulation may exacerbate neuromuscular dysfunction. Management: Longitudinal functional assessment and CK monitoring; withhold ICI and escalate to corticosteroids per ASCO guidance for grade ≥ 2 myositis (51).	(57)
Atezolizumab	Carboplatin + Etoposide	PD-L1 inhibitors combined with chemotherapy widely suppress tumor evasion mechanisms.	Clinical Efficacy: First-line standard regimen for small cell lung cancer. Side Effects: High risk of bone marrow suppression with etoposide.	Used for small cell lung cancer.	Carboplatin may indirectly affect muscle health by impacting overall body function. While the specific effects of etoposide on skeletal muscle are not yet clear, its bone marrow suppression reduces blood cell production, impairing the transport of nutrients and oxygen, and indirectly influencing muscle metabolism and function. When combined with atezolizumab, the immune modulation process may interfere with normal muscle physiology, such as affecting satellite cell activity and reducing the muscle’s self-repair capacity.	Benefit: Standard first-line regimen for extensive-stage SCLC with demonstrated OS benefit. Risk: Myelosuppression impairs oxygen and nutrient delivery to muscle; atezolizumab adds immune myositis risk. Management: Monitor satellite cell-sensitive markers and CK; apply ASCO-aligned graded intervention for immune-mediated muscle adverse events (51).	(58)
Camrelizumab	Cisplatin + Gemcitabine	PD-1 inhibitors enhance chemotherapy-induced immunogenic cell death.	Clinical Efficacy: Rich data in the Chinese population, clear efficacy. Side Effects: May cause reactive capillary proliferation (RCCEP).	Used for nasopharyngeal carcinoma and liver cancer.	Cisplatin may cause bone pain and osteoporosis. Gemcitabine can cause muscle pain, edema with localized pain. When combined with camrelizumab, immune activation and chemotherapy toxicity work together, potentially altering cytokine levels in the muscle microenvironment, affecting the balance between muscle protein degradation and protein synthesis, promoting muscle atrophy, and reducing muscle strength.	Benefit: Substantial clinical evidence in Chinese population for NPC and hepatocellular carcinoma. Risk: Combined cisplatin-gemcitabine muscle toxicity plus camrelizumab-related immune activation may disrupt muscle protein homeostasis; RCCEP monitoring required. Management: CK-based and cytokine-level monitoring throughout treatment; initiate ASCO irAE protocol for immune myositis including corticosteroid therapy at grade ≥ 2 (51).	(59)

In clinical practice, balancing therapeutic efficacy against muscle-related toxicity requires adherence to evidence-based guidelines. The American Society of Clinical Oncology (ASCO) guidelines for managing immune-related adverse events (irAEs) advise clinicians to conduct baseline assessments of serum creatine kinase (CK) levels and muscle-related symptom review before patients start combined chemoimmunotherapy, alongside routine monitoring of muscular manifestations and relevant biomarkers before each treatment cycle and serially during therapy. For patients presenting with grade 2 or higher immune-mediated myositis, clinicians should temporarily hold ICI administration and commence corticosteroid therapy right away. Meanwhile, immediate permanent discontinuation of ICIs paired with multidisciplinary team consultation is mandated for cases featuring myasthenia gravis-like symptoms or markedly elevated CK accompanied by severe muscle involvement. **Table 1** consolidates corresponding risk-benefit analyses and standardized intervention recommendations for reference.

#### 3.1.1 Therapy-specific muscle toxicity of chemotherapeutic agents

Each chemotherapeutic agent triggers distinct skeletal muscle cytotoxicity that superimposes upon the universal inflammatory and metabolic TME-derived injury cascades described above, and the drug-specific myotoxic pathways are analyzed below.

**Figure 3:**
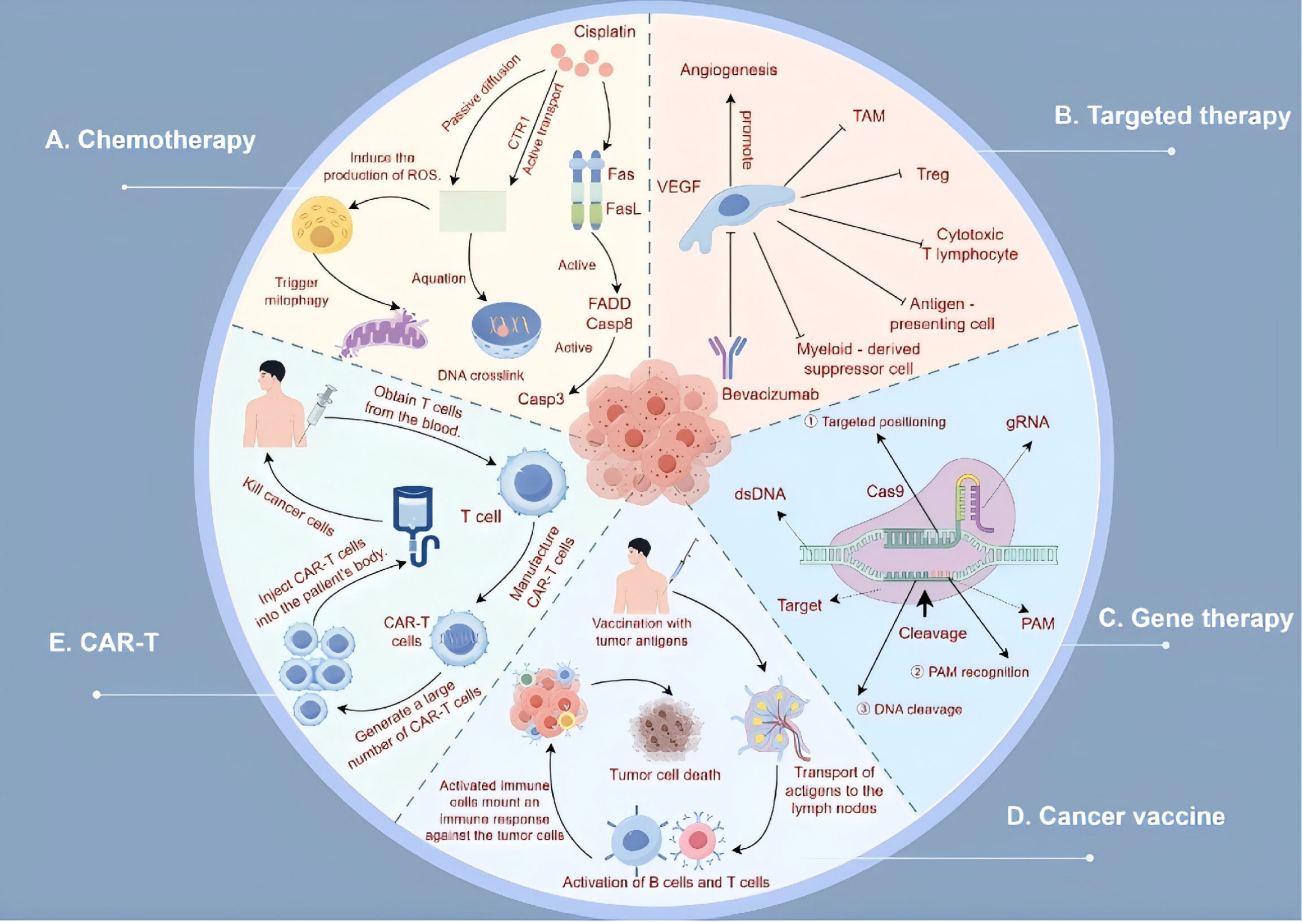
Immunotherapy combined with multiple approaches enhances anti-tumor effects by remodeling the TME. **(A)** The chemotherapeutic drug cisplatin enters cells via passive diffusion and active transport, inducing the generation of reactive oxygen species (ROS), triggering mitochondrial autophagy, causing DNA crosslinking, and activating apoptosis-related signaling pathways. **(B)** Bevacizumab targets vascular endothelial growth factor (VEGF), inhibits angiogenesis, and regulates immune cells in the TME. **(C)** Gene therapy utilizes the CRISPR-Cas9 system for gene editing through targeted positioning, recognition of pre-spacer sequence motifs, and DNA cleavage. **(D)** Cancer vaccines activate B and T cells through tumor antigen vaccination, leading to tumor cell death. **(E)** CAR-T cell therapy involves extracting T cells from the patient’s blood and modifying them into CAR-T cells to recognize and kill tumor cells, triggering an immune response.

Chemotherapy drugs exert cytotoxic effects on tumor cells but can also damage skeletal muscle. Cisplatin-induced DNA damage can trigger oxidative stress, apoptosis, and pro-inflammatory cellular responses (65, 66). In skeletal muscle, cisplatin-associated toxicity is primarily linked to oxidative stress, mitochondrial dysfunction, and the activation of proteolytic and apoptotic pathways. Increased intracellular reactive oxygen species can impair mitochondrial integrity and ATP production, thereby promoting myofiber dysfunction and muscle wasting (67). These cellular processes are summarized in **Figure 3A**. Therefore, cisplatin-related skeletal muscle impairment should be interpreted as metabolic and cellular toxicity rather than a direct neuromuscular blocking effect.

Cyclophosphamide is an alkylating anticancer drug whose mechanism of action in chemotherapy is primarily through its metabolite, phosphoramide mustard, which exerts cytotoxicity by damaging tumor cell DNA and hindering its proliferation (68). Cyclophosphamide reportedly prolongs succinylcholine-induced muscle paralysis by inhibiting pseudocholinesterase. It affects muscle function by inhibiting specific components of the nervous system but does not directly cause muscle paralysis (69).

Fluorouracil (5-FU) is an antimetabolite chemotherapeutic drug that is widely used to treat various cancers. 5-FU is now known to exert its cytotoxic effect by inhibiting thymidylate synthase, interfering with DNA synthesis, preventing the conversion of deoxyuridine to deoxythymidine monophosphate, and inhibiting RNA synthesis (70). It may also induce bone marrow suppression and reduce the white blood cell and platelet counts. In rare cases, 5-FU may damage the kidney and myocardial function or cause neurological side effects, such as cerebellar degeneration and ataxia. 5-FU may indirectly affect muscle control and function owing to its impact on the nervous system (70).

**Figure 4:**
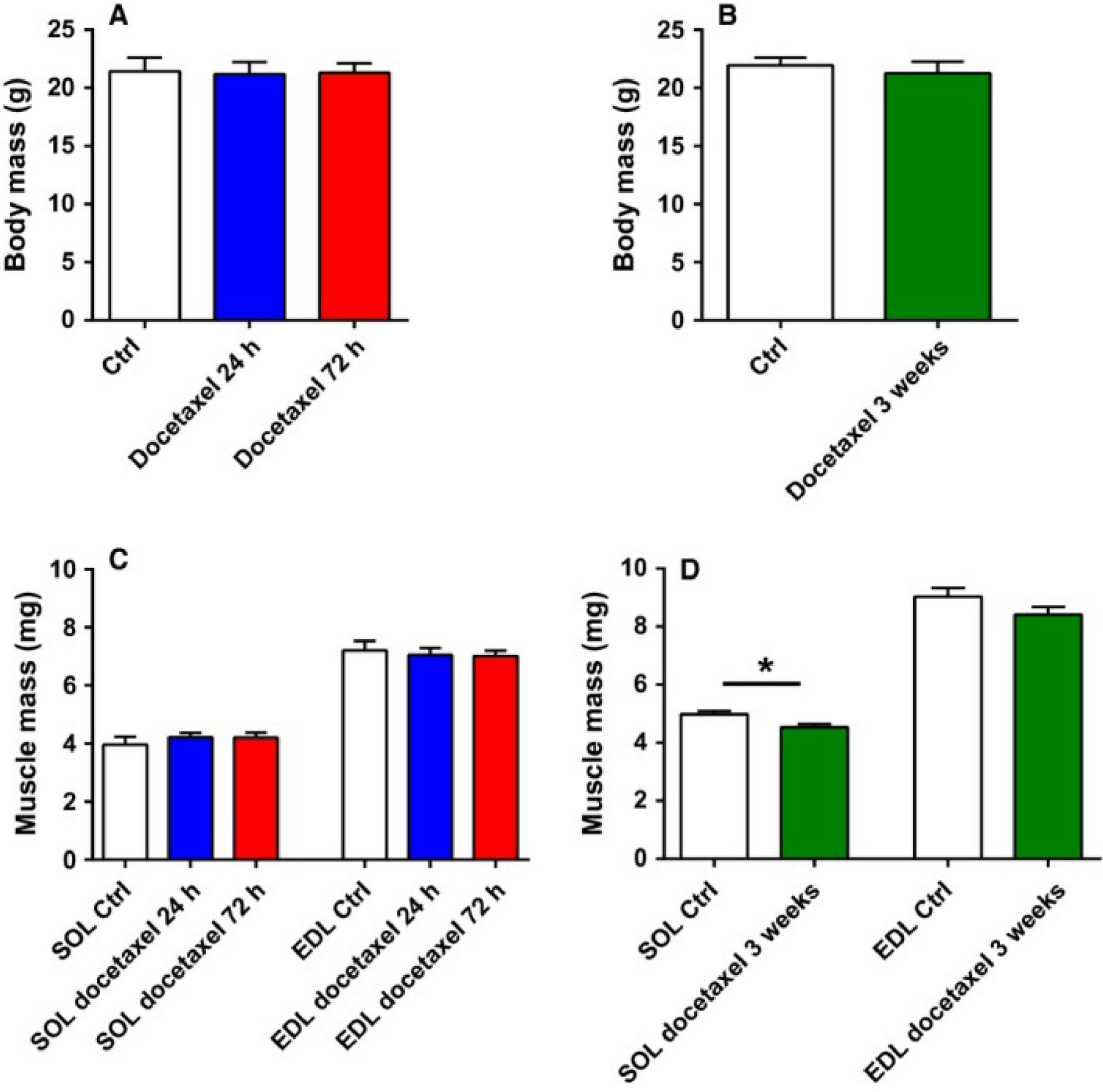
Body mass and muscle mass in healthy mice treated with docetaxel. Body mass after acute treatment (**A**; n = 4 animals) or repeated treatment (**B**; n = 6 animals). Soleus and extensor digitorum longus muscle masses after acute treatment (**C**; n = 7–8 muscles) or repeated treatment (**D**; n = 9–12 muscles). Ctrl, control; SOL, soleus; EDL, extensor digitorum longus; * P < 0.05. Reproduced from Chaillou et al. (80) under the Creative Commons Attribution License.

Doxorubicin, also known as Adriamycin (DOX), is a widely used anticancer drug that inhibits tumor cell growth by binding to DNA and inhibiting topoisomerase II (71). Although DOX exhibits therapeutic efficacy, it can adversely affect skeletal muscle function, and the biological mechanisms behind this phenomenon are complex. DOX-induced oxidative stress originates from massive intracellular ROS overproduction; excessive ROS disrupt calcium homeostasis and mitochondrial integrity, ultimately triggering myocyte apoptosis and muscle catabolism (72). Accumulating evidence confirms that chemotherapeutic agents including cisplatin and doxorubicin share a common ROS-dependent toxic pathway toward skeletal muscle tissue (72). Furthermore, it may disrupt calcium homeostasis within muscle cells, leading to Ca^2+^ overload, which directly affects muscle contraction and relaxation (73). Mitochondria, the cell’s primary energy-producing organelle, may also be damaged by DOX, reducing ATP production and affecting the muscle energy supply (74). DOX may also interfere with autophagy and apoptosis and promote muscle cell death (75). Autophagy is considered a cellular mechanism for the clearance of damaged components; however, DOX may disrupt this process and accelerate cell death (76). DOX may also alter nitric oxide levels and amino acid metabolism, which are crucial for muscle function. Simultaneously, DOX may change the expression of muscle regulatory factors, altering the balance between muscle protein synthesis and degradation, thus affecting muscle mass and function (77). Recent studies have shown that DOX may induce a catabolic response in skeletal muscles due to inflammatory cytokines, particularly TNF, which causes loss of muscle mass through oxidative stress, leading to muscle weakness and fatigue (78). Docetaxel is a taxane chemotherapeutic agent that disrupts microtubule dynamics, thereby inhibiting mitosis and tumor-cell proliferation (79). In healthy mice, neither acute nor repeated intravenous docetaxel treatment significantly altered body mass or ex vivo force production in soleus and extensor digitorum longus muscles. After a single injection, soleus and extensor digitorum longus muscle masses remained unchanged at 24 and 72 h (**Figures 4A, C**). Following three weekly injections, body mass remained unchanged (**Figure 4B**), whereas soleus muscle mass decreased by approximately 9% (P < 0.05). Extensor digitorum longus muscle mass showed a nonsignificant reduction of approximately 7% (P = 0.13; **Figure 4D**). Acute and repeated treatment did not significantly alter absolute force, specific force, fatigue responses, or post-fatigue recovery. These findings suggest that intravenous docetaxel alone may induce modest muscle atrophy without measurably impairing contractile function in healthy mice. However, these results should not be directly extrapolated to tumor-bearing animals or patients with cancer.

In addition, ICIs, including PD-1 monoclonal antibodies, have revolutionized cancer treatment because of their outstanding efficacy (81). While these inhibitors can activate the immune system to fight tumors, they also exert certain effects on skeletal muscle and may cause adverse reactions, among which inflammatory myopathy is one of the most disabling side effects. PD-1 monoclonal antibodies can remove inhibitory signals from T cells, leading to massive infiltration of CD8+ T cells into the muscle tissue (82). Simultaneously, the use of PD-1 monoclonal antibodies can also result in elevated creatine kinase levels (51). Creatine kinase is an essential marker of muscle damage, and its increase indicates that muscle tissue has been damaged to some extent during immunotherapy. When PD-1 monoclonal antibodies are used in combination with chemotherapy drugs, decreased skeletal muscle strength is observed because of the combined effect of chemotherapy drugs on muscle structure and neuromuscular junctions, as well as the dual impact of immune responses triggered by immunotherapy on muscle tissue, which presents a significant challenge to the patient’s muscle function (80).

#### 3.1.2 Dynamic regulatory role of the TME

Chemotherapy induces immunogenic cell death in tumor cells via cytotoxic mechanisms (83). In this context, tumor cells release substances including ATP and HMGB1, which activate the cGAS-STING pathway and promote dendritic cell maturation (84). Dendritic cells are essential for initiating and enhancing anti-tumor immune responses (85). Their maturation allows them to better present tumor antigens to T cells and activate T cells to attack tumor cells (86). However, while chemotherapy induces immunogenic cell death, it recruits numerous myeloid-derived suppressor cells (MDSCs) (84). MDSCs are now understood to harbor strong immunosuppressive functions, secreting cytokines including IL-10, which inhibit T cell activity, thereby weakening the body’s anti-tumor immune response (87). TGF-β in the TME is also a key factor in the combination of immunotherapy and chemotherapy (88). When the concentration of TGF-β in the TME exceeds a certain threshold, it induces an increase in muscle stiffness through the SMAD3 pathway (88), affecting the normal contraction and relaxation of muscles, thereby impacting patient mobility. TGF-β also upregulates PD-L1 expression on the surface of tumor cells. Binding of PD-L1 to the PD-1 receptor on T cells further inhibits T cell activity, weakening the efficacy of PD-1 inhibitors (89).

### 3.2 Immunotherapy combined with targeted therapy

The integration of immunotherapy with targeted therapy represents an innovative cancer treatment strategy that combines ICIs with drugs that target specific molecular pathways (76). These molecularly targeted drugs include anti-angiogenesis drugs and kinase inhibitors (90). Through this combined approach, the aim is to precisely regulate the TME, overcome the immune evasion mechanisms of tumor cells, and subsequently enhance the immune response of the body, leading to more effective tumor destruction (91).

#### 3.2.1 Heterogeneous effects of targeted drugs on skeletal muscle

Unlike the direct cytotoxic muscle damage characteristic of chemotherapy, targeted therapies exert therapy-specific muscle effects primarily through disruption of vascular supply and modulation of intracellular signaling/autophagy pathways.

Targeted therapeutic drugs typically exhibit heterogeneous effects on the skeletal muscle. Bevacizumab targets vascular endothelial growth factor (VEGF), inhibiting angiogenesis and regulating immune cells in the TME (**Figure 3B**) (91). In the TME, it reduces tumor angiogenesis by inhibiting the VEGF signaling pathway, thus inhibiting tumor growth (92). However, clinical studies have found that patients receiving bevacizumab may experience skeletal muscle-related adverse effects, including muscle fatigue and weakness, although the specific mechanisms remain unclear. These effects may be associated with the indirect impact of the drug on skeletal muscle angiogenesis, leading to suboptimal nutritional supply to skeletal muscles. Kinase inhibitors, including sorafenib, are commonly used to treat various solid tumors, but chronic administration can have toxic effects on the skeletal muscle (93). Studies have shown that Sorafenib can lead to a decrease in skeletal muscle mass and strength in mice. The underlying mechanism may involve the regulation of ERK1/2 and GSK3β signaling pathways as well as increased expression of autophagy markers, inducing skeletal muscle atrophy. The effects of different kinase inhibitors on skeletal muscle vary (94). For example, lenvatinib has demonstrated a relatively small negative impact on skeletal muscles (93). In a study involving patients with hepatocellular carcinoma, those treated with sorafenib experienced a more significant decrease in the skeletal muscle mass index, whereas the lenvatinib group showed a smaller reduction (93).

#### 3.2.2 TME remodeling and muscle metabolism interaction

As detailed in the shared pathway framework above, lactate accumulation and MDSC/Treg-mediated immunosuppression constitute universal mechanisms across all combination regimens. In the specific context of targeted therapy combinations, these metabolic perturbations interact with drug-induced vascular remodeling to create compounded muscle metabolic stress (95). CXCL12 secreted by CAFs plays an important role in the TME. By secreting CXCL12, CAFs recruit immunosuppressive cells, promote tumor cell proliferation, migration, and invasion, and induce macrophages to polarize towards the M2 phenotype, further creating an immunosuppressive environment (96). Interestingly, CAFs engage in metabolic crosstalk with tumor cells, supporting oncogenic progression through nutrient provision via metabolic reprogramming (43). In addition, CAFs can increase the production of lactate and pyruvate, thereby providing energy and substrates for tumor cell biosynthesis. This metabolic interaction may also indirectly affect skeletal muscle metabolism, such as causing systemic metabolic disturbances, which influence the nutritional supply and function of skeletal muscles.

### 3.3 Immunotherapy combined with gene therapy

The integration of immunotherapy with gene therapy represents an innovative cancer treatment strategy that aims to enhance the body’s anti-tumor immune response using gene vectors or gene editing technologies. At the same time, by combining ICIs, this approach overcomes the immune suppression caused by tumor cells, thereby enabling more effective tumor resistance (97).

#### 3.3.1 Potential risks of gene vectors on skeletal muscle

The muscle injury profile of gene therapy is mechanistically distinct from both chemotherapy (direct cytotoxicity) and targeted therapy (vascular/signaling disruption). Gene vectors primarily pose risks through immunogenicity-driven muscle inflammation and off-target genomic effects—mechanisms unique to this modality.

Gene vectors used in gene therapy pose potential risks to skeletal muscles (98). Adenoviral vectors are commonly used in gene therapy owing to their efficient capacity to transduce various cell types (99). However, in skeletal muscle gene therapy, adenoviral vectors may trigger immune responses, leading to vector clearance and reduced transduction efficiency (100). Studies have shown that the immune response of the body to adenoviral vectors may involve both humoral and cellular immunity (99). Neutralizing antibodies targeting adenoviral capsid proteins can block the vector from entering cells, whereas cytotoxic T lymphocytes can attack transduced cells, thereby affecting the effectiveness of gene therapy (100). The safety profile of adenoviral vectors involves the potential risk of insertional mutagenesis, which may lead to genomic instability. Gene therapy utilizes the CRISPR-Cas9 system for gene editing, which involves steps such as targeted positioning, recognition of the pre-spacer sequence, and DNA cleavage (**Figure 3C**) (101). In tumor therapy, this technology aims to enhance the body’s anti-tumor immune response; however, when applied to skeletal muscle, it presents potential risks (102). The CRISPR-Cas9 system may induce off-target effects, potentially affecting the normal physiological functions of skeletal muscles (103). In the integration of immunotherapy and gene therapy, it is vital to utilize gene editing technology to enhance immunotherapy effectiveness while also addressing its potential risks to skeletal muscles and exploring safer and more effective gene therapy strategies (104).

#### 3.3.2 Remote regulation of gene expression in the TME

Within the TME, remote regulation of gene expression plays an important role in tumor growth and the efficacy of immunotherapy. Granulocyte-macrophage colony-stimulating factor (GM-CSF) typically has dual functions (105). On one hand, GM-CSF promotes the maturation and activation of dendritic cells, enhancing their antigen-presenting ability and activating T cell-mediated anti-tumor immune responses (106). In animal experiments, administration of GM-CSF was found to increase the abundance and activity of dendritic cells in the TME, improve T cell recognition, and eliminate tumor cells (107). In some cases, GM-CSF may promote tumor cell growth and metastasis, which may be related to the regulation of the cytokine network within the TME and immune cell infiltration (105). Hypoxia-inducible factor-1α (HIF-1α) plays a key role in angiogenesis in the TME (108). Current evidence suggests that tumor tissues are often in a hypoxic state, which induces upregulation of HIF-1α expression (109). HIF-1α promotes tumor angiogenesis by regulating the expression of multiple target genes, such as VEGF, thereby providing tumors with nutrients and oxygen to support tumor growth and metastasis. HIF-1α also regulates metabolic reprogramming of tumor cells, enhancing their survival ability in hypoxic environments (110). In addition, HIF-1α can affect the function of immune cells in the TME, including promoting immune evasion and inhibiting T-cell activity (108).

### 3.4 Immunotherapy combined with tumor vaccines

The integration of immunotherapy with tumor vaccines represents an innovative strategy for cancer treatment. Its fundamental principle involves utilizing tumor vaccines to activate the host’s specific T cell response while using ICIs to enhance the body’s immune memory against tumors, thereby more effectively targeting tumor cells and preventing tumor recurrence.

#### 3.4.1 Vaccine-induced cross-immunity

The muscle effects of tumor vaccines differ fundamentally from those of chemotherapy, targeted therapy, and CAR-T approaches. Vaccine-specific muscle involvement is primarily transient and mild, driven by adaptive immune activation rather than direct cytotoxicity, metabolic disruption, or cytokine storm. While the shared TME pathways remain relevant as background, they are not the dominant mechanism of vaccine-associated muscle symptoms.

Vaccine-induced cross-immunity is an important strategy in tumor immunotherapy. Cancer vaccines activate B and T cells through tumor antigen administration, leading to tumor cell death (**Figure 3D**) (110). Personalized vaccines are tailored based on the genetic mutation profile of a patient’s tumor and specifically activate the patient’s immune system to recognize and attack tumor cells. For example, by performing whole-exome sequencing of tumor tissue, tumor-specific mutations have been identified, and personalized vaccines have been designed based on these mutations, which enhances the specificity and efficacy of the vaccine (111). Adjuvants, such as CpG, play a key role in enhancing the immunogenicity of vaccines (110). CpG is an oligodeoxynucleotide that stimulates the innate immune response by activating Toll-like receptor 9 (TLR9), promoting dendritic cell maturation and activation, and enhancing antigen presentation, thereby increasing the intensity of the immune response induced by the vaccine. Studies have shown that in animal models, tumor vaccines using CpG as an adjuvant significantly enhance vaccine-specific antibodies and T cell responses, improving the inhibitory effect on tumors. Different types of adjuvants used in combination with vaccines can induce various immune responses such as Th1 or Th2 responses. Through rational adjuvant selection, the vaccine’s immune effects can be optimized, inducing broader cross-immunity and improving its ability to kill different tumor cells (112).

#### 3.4.2 Immune memory formation: the role of memory T cells and fibroblasts

Within the TME, the formation of immune memory is crucial for long-term tumor control. Sustained activation of memory T cells is a key step in the formation of immune memory. When a tumor vaccine activates the immune system, some T cells differentiate into memory T cells, which can persist in the TME and rapidly reactivate upon encountering tumor antigens again, thereby exerting anti-tumor effects. For example, in certain tumor models, memory T cells induced by vaccines can reside in tumor tissues for extended periods, providing continuous immune surveillance of tumor cells (113). Fibroblasts in the TME play a role in fibrosis as they secrete ECM components that promote the formation of tumor stromal fibrosis. However, this fibrotic process has a complex effect on the formation of immune memory. Fibrosis can provide a physical barrier for tumor cells, hindering the infiltration of immune cells and their attack on tumor cells. However, some cytokines and chemokines secreted by fibroblasts can regulate the recruitment and activation of immune cells, influencing the formation and function of memory T cells. For example, CXCL12 secreted by fibroblasts can recruit Tregs and suppress antitumor immune responses, which is detrimental to the formation and maintenance of immune memory. Therefore, understanding the mechanistic role of fibroblasts in the TME is of great significance for optimizing immunotherapy strategies and promoting immune memory formation (114).

### 3.5 Immunotherapy combined with CAR-T cell therapy

Immunotherapy combined with CAR-T cell therapy represents a state-of-the-art cancer treatment strategy designed to significantly enhance anti-tumor efficacy (115). This approach synergistically combines CAR-modified T cells with ICIs, leveraging the dual mechanism of targeting tumor-specific antigens and eliminating immune suppression, thus markedly potentiating the therapeutic effect against tumors.

#### 3.5.1 Dual effects of CAR-T cells on skeletal muscle

CAR-T cell therapy presents unique muscle toxicity mechanisms not shared by chemotherapy, targeted therapy, or vaccines: (1) direct “on-target, off-tumor” immune attack on muscle cells expressing cross-reactive antigens, and (2) cytokine release syndrome (CRS)-mediated systemic metabolic storm driven by massive and rapid CAR-T cell activation. These therapy-specific effects are superimposed upon the shared TME-muscle pathways described in the chapter introduction.

In CAR-T cell therapy, T cells are extracted from a patient’s blood and modified with CARs to recognize and kill tumor cells, thereby triggering an immune response (**Figure 3E**) (116). In cancer treatment, CAR-T cell therapy exerts a robust anti-tumor effect by targeting tumor-specific antigens. However, it has dual effects on skeletal muscle, including direct toxicity and indirect metabolic interference (116). In terms of direct toxicity, CAR-T cells may mistakenly recognize certain antigens on the surface of skeletal muscle cells, leading to direct immune attack and consequent damage to the skeletal muscle. Studies have reported that some patients undergoing CAR-T cell therapy develop symptoms such as myositis and muscle weakness, which may be related to the direct toxicity of CAR-T cells on skeletal muscle. CAR-T cell therapy can exert indirect metabolic effects, potentially disrupting systemic homeostasis by impairing skeletal muscle metabolism and functional capacity. For example, CAR-T cell therapy may lead to cytokine release syndrome, which causes a systemic inflammatory response that affects skeletal muscle energy metabolism and protein synthesis (117). Moreover, CAR-T cell therapy may disrupt insulin signaling pathways, interfering with the ability of skeletal muscle to uptake and utilize glucose, leading to metabolic abnormalities within skeletal muscle (118). Moreover, the lymphodepleting chemotherapy regimen administered prior to CAR-T cell infusion may exert an additional toxic effect on skeletal muscles, exacerbating muscle damage (119).

#### 3.5.2 Interaction between TME and muscle immunity

Current evidence suggests a complex interaction between the TME and muscle immunity. CAR-T cells can reportedly undergo activation in the TME and exert a powerful anti-tumor immune effect. CAR-T cells specifically recognize tumor antigens and activate their immune-killing functions to attack tumor cells. In the TME, CAR-T cells can secrete various cytokines, including interferon-γ, which further activates other immune cells and enhances the anti-tumor immune response (120). However, stromal cells in the TME, including CAFs, can affect CAR-T cell function and muscle immunity through pro-fibrotic reactions. CAFs secrete large amounts of ECM components, leading to tumor stromal fibrosis and forming a physical barrier that hinders the infiltration and migration of CAR-T cells towards tumor cells. At the same time, CAFs can also secrete immunosuppressive factors such as TGF-β, which inhibit the activity of CAR-T cells and weaken the anti-tumor immune response (121). In addition, there is a reciprocal relationship between stromal cells and muscle immunity. The metabolic products and cytokines secreted by stromal cells may affect the immune status of muscle tissue, whereas changes in muscle immune cells may regulate the function of stromal cells. This interaction has a significant impact on the efficacy of CAR-T cell therapy and the health status of skeletal muscles.

### 3.6 Clinical confounding variables and population heterogeneity

Beyond the pharmacological and immune injury mechanisms outlined in the preceding content, clinical intervention strategies and patients’ intrinsic biological traits profoundly modulate the severity of skeletal muscle dysfunction triggered by combination immunotherapy. These confounding factors have been largely overlooked in prior reviews and represent critical variables for accurate assessment of treatment-related myotoxicity.

#### 3.6.1 Corticosteroid use: the muscle metabolism paradox in irAE management

Systemic corticosteroids represent the first-line clinical intervention for severe immune-related adverse events (irAEs) such as immune myositis triggered by combination immunotherapy, but they bring complicated confounding effects on skeletal muscle homeostasis. On the therapeutic side, high-dose glucocorticoids rapidly suppress NF-κB and pro-inflammatory cytokine (TNF-α, IL-6) release to relieve autoimmune muscle injury. However, prolonged or repeated corticosteroid exposure strongly activates the muscle ubiquitin-proteasome axis through the glucocorticoid receptor (GR)→FoxO3 transcriptional pathway, directly upregulating atrophy markers MuRF1 and Atrogin-1, and inhibits satellite cell proliferation and myogenic differentiation, eventually leading to steroid-induced myopathy (122).

Clinicians therefore face a contradictory trade-off: steroids alleviate immunogenic muscle inflammation yet simultaneously aggravate long-term muscle mass loss and weakness. This paradox creates significant diagnostic confusion—post-treatment muscle dysfunction may originate from the immunotherapy itself, corticosteroid-induced myopathy, or their superimposition, making accurate attribution of muscle adverse events extremely challenging. Rational tapering regimens are therefore required to minimize secondary muscle atrophy while controlling irAE progression. Future clinical studies should incorporate corticosteroid cumulative exposure as a covariate in analyses of immunotherapy-related muscle outcomes, and establish differential diagnostic criteria (e.g., muscle biopsy for inflammatory infiltration patterns, specific autoantibody panels) to distinguish immune myositis from steroid myopathy (123).

#### 3.6.2 Age-related differences in muscle vulnerability

Patient age is a critical intrinsic variable that determines the severity of immunotherapy-associated skeletal muscle dysfunction. Elderly individuals (≥65 years) possess inherently impaired satellite cell activity, weakened antioxidant defense systems, and diminished mTORC1-mediated anabolic responsiveness (“anabolic resistance”). Upon exposure to elevated TGF-β and pro-inflammatory cytokines generated during combination immunotherapy, elderly patients develop more severe, often irreversible muscle atrophy and cachexia compared to younger patients. Additionally, age-related mitochondrial DNA mutation accumulation and respiratory chain dysfunction render aged muscle fibers more susceptible to the additive mitochondrial toxicity of chemotherapeutic agents (cisplatin, DOX). The immunosenescence phenotype (“inflammaging”), characterized by chronically elevated baseline IL-6 and TNF-α levels, further amplifies cytokine storm-mediated muscle damage when superimposed upon treatment-induced immune activation (124).

Clinical evidence indicates that elderly patients receiving combination chemo-immunotherapy experience significantly higher incidence and severity of muscle function decline, reduced treatment tolerance, and impaired recovery capacity. These patients should be identified as a high-risk population for muscle adverse events, warranting more conservative dosing strategies and intensified muscle function monitoring.

#### 3.6.3 Sex-related differences in muscle response

In terms of sex differences, endogenous estrogen signaling exerts anti-catabolic effects by inhibiting FoxO3/MuRF1 cascades through estrogen receptor-β (ERβ), so premenopausal female patients generally display milder muscle wasting after combination therapy. However, postmenopausal women lose this protective effect due to estrogen decline, rendering them more susceptible to cytokine-driven muscle atrophy. In contrast, male patients benefit from testosterone-mediated activation of PI3K/Akt/mTOR anabolic signaling but are more prone to TGF-β-mediated muscle fibrosis and persistent muscle stiffness. Notably, prostate cancer patients receiving androgen deprivation therapy (ADT) lose androgenic muscle protection entirely, facing substantially elevated myotoxicity risk during subsequent combination immunotherapy (125).

Sex-based differences in muscle fiber composition further modulate vulnerability patterns: males possess a higher proportion of type II (fast-twitch, glycolytic) fibers that are preferentially susceptible to ubiquitin-proteasome-mediated atrophy, whereas females have relatively more type I (slow-twitch, oxidative) fibers with stronger anti-catabolic capacity. Future clinical muscle monitoring and protective interventions should adopt age- and sex-specific cut-off standards to improve individualized management.

#### 3.6.4 Fiber-type-specific vulnerability and satellite cell dysfunction

Skeletal muscle exhibits heterogeneous vulnerability to therapy-induced damage. Fast-twitch glycolytic fibers (type II; e.g., EDL, tibialis anterior) are more susceptible to cytokine-mediated atrophy and oxidative injury due to higher FoxO3 activity and greater reliance on the ubiquitin-proteasome degradation pathway. In contrast, slow-twitch oxidative fibers (type I; e.g., soleus) display stronger anti-catabolic capacity owing to enhanced mitochondrial antioxidant defenses and lower sensitivity to STAT3/NF-κB-driven proteolysis (32).

Impaired satellite cell proliferation further restricts muscle regenerative potential after immune-related myotoxicity. Sustained TGF-β and TNF-α signaling—both of which are chronically elevated during combination immunotherapy—blocks satellite cell activation, inhibits myogenic differentiation (via Pax7 downregulation and myogenin suppression), and impairs the muscle’s intrinsic self-repair capacity. This regenerative failure transforms acute, potentially reversible muscle injury into chronic, progressive muscle wasting, representing a critical but underappreciated contributor to long-term functional decline in cancer patients receiving combination immunotherapy (126).

Collectively, glucocorticoid intervention, chronological age, sex hormone profiles and inherent fiber heterogeneity jointly determine individual muscle injury susceptibility under combined immunotherapy. Integrating these confounding factors into clinical risk stratification can realize individualized skeletal muscle protection and toxicity monitoring schemes.

Having characterized the mechanisms of injury and the clinical variables that modulate their severity, the accurate measurement of these effects in patients requires a robust and sensitive assessment framework, which is addressed in the following section.

## 4 Assessment methods of skeletal muscle biomechanics and TME in tumor immunotherapy

Prior reviews have described individual assessment modalities for cancer-related muscle evaluation in isolation; this review uniquely integrates their comparative sensitivity profiles for early irAE detection and, proposes a structured framework for incorporating multimodal muscle endpoints into immunotherapy clinical trial design. Accurate and timely assessment of skeletal muscle biomechanical changes during combination immunotherapy is essential for early identification of muscle adverse events, treatment plan optimization, and rehabilitation guidance. This section reviews traditional and emerging evaluation techniques, critically appraises their respective advantages, limitations, and sensitivity for early detection, and discusses their integration into clinical trial design.

### 4.1 Traditional biomechanical assessment techniques

As the field of tumor immunotherapy continues to develop, traditional methods for assessing skeletal muscle characteristics remain the key to analyzing changes in skeletal muscle biomechanics.

#### 4.1.1 Handheld dynamometry

During muscle strength assessment, handheld dynamometers are commonly used because they are easy to operate and can be used for bedside measurements, which are particularly important for cancer patients with mobility issues (127). During tumor immunotherapy, the regular use of a handheld dynamometer to measure the strength of specific muscle groups can provide a clear trend of muscle strength changes. For example, during immunotherapy for patients with breast cancer, measuring upper limb muscle strength can help identify changes in muscle function caused by treatment (128). However, owing to subjective factors, such as the angle of force application and strength control by the tester, the measurement results may be biased. Therefore, when applying this method clinically, standard training of testers is necessary to minimize subjective errors and ensure the reliability of the measurement results.

The advantages of handheld dynamometry include its low cost, portability and compatibility with bedside testing; the technique imposes minimal demands on patient cooperation and is well-suited to repeated longitudinal monitoring of fatigued patients with malignant tumors. However, its measurements are subject to variability tied to the operator, and the method lacks sufficient sensitivity to identify subclinical or incipient muscle weakness. It cannot distinguish whether muscle weakness arises from neural or myogenic origins, and a ceiling effect is observed when assessing individuals with greater muscle strength. Overall, this tool demonstrates moderate sensitivity for early detection: while it is capable of identifying functional strength reductions of roughly 10-15%, it often fails to capture subtle pre-clinical alterations in muscle quality or tissue composition that emerge before measurable losses in overt muscle strength (129).

#### 4.1.2 Dual-energy X-ray absorptiometry and computed tomography

Dual-energy X-ray absorptiometry (DXA) and computed tomography (CT) are the most widely used direct methods for assessing muscle mass in medical practice. DXA distinguishes muscle, fat, and bone components based on differences in their X-ray absorption, with the advantages of low radiation exposure, high accuracy, and strong reliability, making it an ideal tool for muscle mass assessment. It is widely used in the assessment of whole-body muscle mass, diagnosis of osteoporosis, and muscle wasting syndromes, as well as in large-scale screenings and clinical evaluations (130). In the context of tumor immunotherapy, DXA provides detailed information regarding a patient’s muscle mass, allowing the monitoring of changes in muscle mass during treatment and helping to identify adverse effects such as muscle loss. However, DXA equipment is expensive and the procedure involves a certain level of radiation exposure. For patients with cancer who require frequent monitoring and are physically weakened, the potential harm from cumulative radiation effects should be carefully considered.

DXA offers excellent reproducibility with a coefficient of variation typically below 2%, a low radiation dose per scan, and validated reference databases for sarcopenia diagnosis, enabling both whole-body and regional compartmental analyses. Its principal limitation is an inability to differentiate intramuscular fat infiltration from lean tissue and its provision of two-dimensional projection data rather than volumetric measurements. More critically, DXA is insensitive to early functional deterioration that precedes detectable mass loss, meaning that clinically relevant muscle injury may already be established before the instrument signals an abnormality. Its early detection sensitivity is therefore considered low to moderate in the context of immunotherapy-related muscle injury (131).

In contrast, CT can more precisely differentiate between various tissue components in the body and can use algorithms to calculate the volume and mass of these tissues. It is considered the gold standard for muscle mass assessment, particularly when detailed information on muscle distribution and morphology is required. For example, in the preoperative evaluation of bladder cancer patients, CT imaging not only enables visualization of tumor lesions and distant metastases but also simultaneously evaluates muscle mass (132).

CT surpasses DXA by enabling Hounsfield unit-based quantification of muscle density, which reflects myosteatosis—intramuscular fat infiltration associated with functional impairment and poor prognosis. Routine oncological staging CT scans provide an opportunity for opportunistic sarcopenia screening without additional radiation exposure, a major practical advantage in cancer populations already undergoing frequent imaging. The drawback is the higher radiation dose with dedicated scanning protocols, the requirement for specialized segmentation software, and the cost and logistical barriers that preclude its use as a frequent monitoring tool. For early detection, CT muscle density measurement offers moderate-to-high sensitivity, as qualitative tissue changes such as increased fat infiltration and reduced density can precede significant reductions in muscle cross-sectional area (133).

Although both DXA and CT are direct assessment methods, DXA is more commonly used in routine clinical evaluations because of its broad applicability, whereas CT is reserved for situations that require high resolution. The choice of the appropriate method should be guided by the intended assessment purpose and specific condition of the patient. At the molecular level, muscle mass can be assessed by isolating muscle fibers to evaluate their fundamental mechanical properties. Transparent single muscle fibers can be prepared, and upon calcium activation, they provide a straightforward understanding of the functionality of contractile proteins. The key measurement indicators include the maximum strength, specific force, and unloaded shortening velocity.

#### 4.1.3 Isokinetic testing

Isokinetic testing offers distinct advantages in evaluating muscle contractility as it allows for precise control of the speed of muscle contraction, resulting in a comprehensive and objective assessment of muscle function across varying speeds (134). In the intricate domain of tumor immunotherapy and the TME, this characteristic is of significant importance. For instance, isokinetic testing in melanoma patients can assess the effects of immunotherapy on muscle contraction at different movement speeds, thereby establishing a basis for the development of personalized rehabilitation plans. Isokinetic testing equipment is complex and requires a high level of technical expertise from the operator, which limits its widespread adoption in primary healthcare settings (127).

Isokinetic dynamometry provides objective, velocity-specific torque measurements across the full range of joint motion, eliminates effort variability through constant-velocity resistance, and assesses both concentric and eccentric muscle function with high reproducibility, typically yielding intraclass correlation coefficients above 0.90. These properties make it well-suited for reliable longitudinal tracking of muscle functional decline during immunotherapy cycles. Its main limitations are the high cost of specialized equipment, the requirement for trained operators, the inability to use the device at the bedside, and the need for a sufficient degree of patient mobility and cooperation that may not always be achievable in debilitated cancer patients. For early detection, isokinetic testing demonstrates high sensitivity, capturing subtle reductions in peak torque and power at specific angular velocities before patients report subjective weakness or before mass-based imaging detects structural change (129).

#### 4.1.4 Ultrasound elastography

During tumor immunotherapy, ultrasound elastography enables real-time monitoring of changes in muscle stiffness. For instance, the effects of TME alterations on muscle stiffness can be evaluated in colorectal cancer patients receiving immunotherapy. This method is noninvasive, user-friendly, and offers real-time imaging capabilities. However, the accuracy of deep muscle measurements requires enhancement owing to the ultrasound signal attenuation. Therefore, it is crucial to integrate this method with other techniques to comprehensively assess changes in deep muscle stiffness.

Ultrasound elastography is non-invasive, radiation-free, portable, and capable of providing quantitative stiffness metrics—such as shear wave velocity and Young’s modulus—in real time, with no cumulative harm from repeated assessments. It is particularly valuable for detecting fibrotic remodeling and inflammatory stiffening within muscle tissue, which are processes directly driven by TME-derived TGF-β and cytokine cascades characteristic of combination immunotherapy. Its limitations include operator-dependent probe positioning, reduced penetration depth for deep muscle groups, susceptibility to confounding by subcutaneous edema and adipose tissue common in cancer patients, and the current absence of standardized protocols or oncology-specific normative databases. In terms of early detection sensitivity, elastography holds moderate-to-high promise: stiffness changes reflecting early inflammatory infiltration or incipient fibrosis can be detected before structural atrophy is visible on conventional imaging, positioning this tool as a potentially valuable early indicator of TME-driven muscle remodeling (135).

Traditional methods can effectively analyze alterations in skeletal muscle biomechanics within the intricate framework of tumor immunotherapy and TME. Changes in muscle strength may be associated with muscle inflammation or nerve damage resulting from tumor immunotherapy, and may also be influenced by cytokines present in the TME. Additionally, variations in muscle mass are linked to factors such as nutritional support from the TME and immune cell infiltration, with immunotherapy potentially affecting muscle mass indirectly through modulation of the TME. Fluctuations in muscle contractility and stiffness are closely tied to inflammation and fibrosis within the TME, and are affected by both the direct and indirect effects of immunotherapy agents (136). The alterations in skeletal muscle biomechanics identified by these traditional methods can be further analyzed for their correlation with the outcomes of tumor immunotherapy and changes in the TME, providing valuable insights for clinical treatment and rehabilitation.

### 4.2 Emerging detection and evaluation techniques

**Figure 5:**
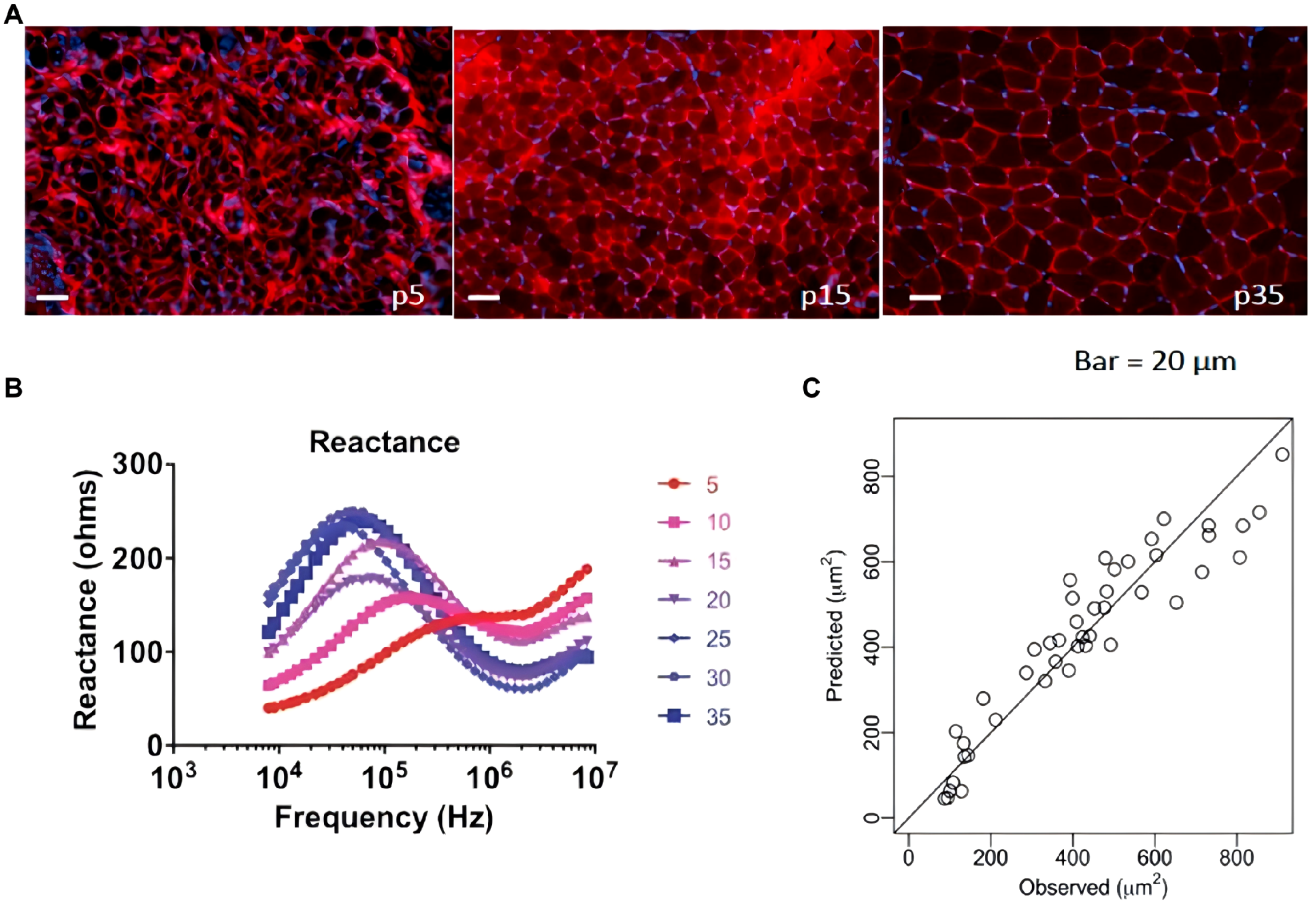
Prediction of myofiber size using electrical impedance myography (EIM). **(A)** Representative immunofluorescence images of gastrocnemius muscle sections from mice at postnatal days 5, 15, and 35. Sections were stained for collagen VI (red, myofiber boundaries) and DAPI (blue, nuclei). Myofiber cross-sectional area increases with postnatal maturation; scale bar, 20 μm. **(B)** Multifrequency reactance spectra recorded from mice aged from postnatal day 5 to postnatal day 35. The reactance curves shift progressively with advancing age. **(C)** Scatter plot comparing myofiber cross-sectional area predicted using LASSO regression based on the full multifrequency EIM dataset with histologically measured values. Data points cluster near the identity line, demonstrating strong predictive performance of the EIM model. Adapted from Kapur et al. (138) with permission.

Electrical impedance myography is an innovative technology that offers distinct advantages for the assessment of skeletal muscle biomechanics. This non-invasive diagnostic method evaluates muscle function and mass by analyzing the impedance characteristics of the muscle tissue. During the EIM examination, a low-level, imperceptible current (approximately 400 µA) was applied across multiple frequencies ranging from 1 kHz to 1 MHz. The voltage measured through the two sensing electrodes is subsequently utilized to derive parameters, including the muscle phase angle, reactance, and resistance (137). The EIM reflects muscle health and encompasses aspects such as atrophy, fibrosis, and fat infiltration. It is sensitive to changes in muscle health status owing to factors such as growth, training cessation, and obesity. Furthermore, the EIM is employed in the assessment of neuromuscular diseases, including muscular dystrophy and ALS. Kapur et al. conducted a study investigated the potential of EIM in estimating muscle fiber size using standard predictive modeling methods. Wild-type immature mice of different ages were selected (**Figure 5A**). Given the small size of the mice, a needle electrode array was used to perform EIM on the gastrocnemius muscle, followed by quantification of the muscle fiber size. The multifrequency impedance-reactance characteristics changed as the mice grew and developed (**Figure 5B**). Advanced data analysis methods, such as minimum absolute contraction and selection operators, used the240 EIM parameters obtained from each measurement to accurately estimate the average muscle fiber size for all groups (explained variance of approximately 90%; **Figure 5C**). In tumor immunotherapy, EIM can detect subtle changes in the muscle early, providing a basis for timely adjustments in treatment plans. For example, during the early stages of immunotherapy in leukemia patients, EIM can detect small changes in the electrical properties of muscle tissue, whereas traditional methods may not yet show obvious abnormalities. This helps with early intervention and prevents further muscle function deterioration (139). EIM has great potential for monitoring the impact of TME changes on skeletal muscles. Alterations in cytokines, inflammatory factors, and other elements in the TME can affect the electrical properties of muscle tissue. When the TME is in an inflammatory state, muscle tissue impedance changes, and the EIM can detect these changes to reflect the interaction between the TME and skeletal muscle (140). Emerging technologies provide more comprehensive and precise data for studying the relationship between tumor immunotherapy, TME, and skeletal muscle biomechanics. EIM can not only measure muscle impedance parameters but also analyze impedance changes under different frequencies of current to obtain information about the microscopic structure and function of the muscle (141). Combined with traditional methods, it can reveal the complex internal relationships between the three aspects from multiple perspectives. For example, combining EIM with DXA can provide insights into both muscle mass changes and microscopic structural changes, offering a more comprehensive evaluation of the influence of tumor immunotherapy on skeletal muscle (142). At the same time, EIM data can be correlated with various indicators in the TME (such as cytokine levels and immune cell counts), offering new insights into the dynamic relationship between tumor immunotherapy, TME, and skeletal muscle biomechanics, and providing strong support for the development of more effective cancer treatment strategies and muscle protection measures.

EIM combines strong clinical practicality with high mechanistic sensitivity: the entire measurement is completed in under five minutes, requires no specialized infrastructure beyond a portable device, and generates no radiation burden, enabling frequent point-of-care and potentially home-based longitudinal monitoring throughout the immunotherapy course. Its multifrequency signal analysis provides composite tissue characterization spanning edema, fibrosis, fat infiltration, and fiber architecture that no single traditional modality can simultaneously capture (143). The limitation of EIM lies principally in the current absence of universally standardized electrode configurations and oncology-specific normative reference databases, alongside susceptibility to confounding from skin temperature, subcutaneous fat distribution, and limb edema, all of which are common complications in cancer patients. Large-scale validation in immunotherapy cohorts remains needed before routine clinical adoption (128).

Nevertheless, EIM currently demonstrates the highest early detection sensitivity among available non-invasive tools: the phase angle parameter, in particular, can detect sub-clinical micro-architectural disruptions reflecting early inflammatory infiltration or tissue remodeling before any functional strength loss or imaging-detectable mass reduction is apparent, positioning EIM as a leading candidate for early biomarker integration in immunotherapy muscle monitoring protocols.

### 4.3 Integration of skeletal muscle assessment into clinical trial design

The assessment modalities described above have largely been deployed in observational oncology settings. However, skeletal muscle dysfunction is increasingly recognized as a clinically meaningful endpoint that warrants systematic incorporation into prospective immunotherapy trial frameworks. This section outlines practical recommendations for integrating muscle biomechanical assessment into trial design (130).

Assessment timepoints should be structured to capture the full trajectory of treatment-related muscle change. A comprehensive baseline evaluation—combining CT or DXA-based muscle mass quantification, functional testing via HHD or isokinetic dynamometry, EIM, and serum biomarkers including creatine kinase, aldolase, IL-6, and myostatin—should be completed before immunotherapy initiation to establish individual reference values. Serial functional assessments using HHD and EIM at every two to four treatment cycles enable detection of early subclinical decline with minimal patient burden, while CT-based muscle analysis can be opportunistically incorporated into routine restaging scans without additional radiation. Post-treatment and follow-up assessments at three, six, and twelve months should incorporate DXA or CT for mass quantification, isokinetic testing to characterize functional recovery trajectories, and elastography to evaluate residual fibrosis (132).

Given that no single modality captures all dimensions of immunotherapy-related muscle injury, trials should adopt composite muscle endpoints integrating structural indices such as CT-derived skeletal muscle index and muscle density, functional measures including isokinetic peak torque and six-minute walk distance, tissue quality parameters such as EIM phase angle and elastography shear wave velocity, and circulating biomarkers. This multimodal approach enhances early detection sensitivity while providing mechanistic insight into whether observed deterioration reflects inflammatory, metabolic, fibrotic, or neurogenic pathophysiology—information that is directly actionable for clinical decision-making.

Pre-specified stratification is essential given the population heterogeneity described in Section 3.6. Trials should stratify by age (below versus above 65 years), sex and hormonal status including postmenopausal status and androgen deprivation therapy exposure, baseline sarcopenia status using validated skeletal muscle index cut-offs, and cumulative corticosteroid exposure during treatment. Such stratification enables identification of high-risk subgroups for whom intensified monitoring or early protective intervention—such as resistance exercise or nutritional support—may be warranted.

A tiered monitoring algorithm can optimize resource utilization across diverse trial settings. All patients should undergo HHD grip strength measurement, serum CK assessment, and patient-reported muscle symptom questionnaires at every cycle as a first-tier screen. Patients who trigger threshold abnormalities or who belong to high-risk strata should proceed to a second tier encompassing EIM, ultrasound elastography, and an expanded biomarker panel. Confirmed or severe muscle dysfunction should prompt a third tier including CT volumetric analysis, isokinetic testing, specialist rheumatology or rehabilitation referral, and consideration of diagnostic muscle biopsy to distinguish immune myositis from steroid-induced myopathy. Systematic adoption of such structured protocols in immunotherapy trials will generate prospective evidence linking biomechanical endpoints to treatment outcomes, establish validated intervention thresholds, and support regulatory recognition of muscle function as a co-primary or key secondary endpoint alongside tumor response and survival measures (144).

## 5 Future research directions and prospects

Building upon the mechanistic, clinical, and assessment frameworks established in the preceding sections, this chapter identifies critical unresolved questions and proposes research priorities that distinguish the future agenda of this field from those outlined in our prior review (4). Whereas earlier work called broadly for further mechanistic investigation of ICI-induced muscle injury, this review highlights the need for intervention translation, precision biomarker validation, and population-stratified clinical trial design as the defining next steps.

### 5.1 Muscle-protective intervention strategies

The mechanistic understanding of combination immunotherapy-induced skeletal muscle dysfunction accumulated in this review provides a direct basis for designing targeted protective interventions. Despite growing recognition of treatment-related muscle wasting as a major determinant of patient quality of life and treatment tolerance, muscle-protective strategies remain systematically underutilized in oncological practice. This section consolidates evidence-based and emerging intervention approaches, with emphasis on their optimal timing relative to immunotherapy cycles (145).

Resistance exercise represents the most robustly evidenced intervention for preserving skeletal muscle mass and function in cancer patients. Progressive resistance training activates the PI3K/Akt/mTOR anabolic signaling pathway, counteracting the FoxO3/MuRF1/Atrogin-1 catabolic cascade driven by TME-derived cytokines. Clinical trials in patients receiving chemotherapy-based regimens have demonstrated that supervised resistance exercise initiated prior to or concurrent with treatment significantly attenuates skeletal muscle index decline, preserves grip strength, and reduces fatigue severity compared to standard care. In the context of combination immunotherapy, the timing of exercise relative to treatment cycles is critical: moderate-intensity resistance training during the inter-cycle interval (days 3–14 post-infusion) appears better tolerated and more anabolically effective than exercise immediately following ICI administration, when cytokine-mediated muscle inflammation may be at its peak. Future trials should prospectively investigate the optimal frequency, intensity, and timing of resistance exercise protocols specifically calibrated to combination immunotherapy regimens, with stratification by fiber-type composition, age, and sex as described in Section 3.6.

Nutritional support constitutes a complementary pillar of muscle protection, targeting the anabolic insufficiency and catabolic excess that characterize combination immunotherapy-associated muscle wasting. Adequate protein intake—at minimum 1.2-1.5 g/kg/day, and up to 2.0 g/kg/day in sarcopenic or elderly patients—is essential to support muscle protein synthesis against background ubiquitin-proteasome activation. Leucine-enriched essential amino acid supplementation has demonstrated particular efficacy in stimulating mTORC1-dependent protein synthesis in cancer cachexia models. Omega-3 fatty acids, particularly eicosapentaenoic acid, suppress TNF-α and IL-6 production and NF-κB activation, thereby attenuating the inflammatory component of muscle catabolism. Vitamin D supplementation supports satellite cell function and myogenic differentiation—both impaired by sustained TGF-β exposure during immunotherapy. Nutritional interventions should be initiated at treatment commencement rather than after clinically detectable muscle loss, since the window for reversible intervention narrows substantially once myosteatosis and fibrosis are established (146).

Anti-cachexia pharmacological agents represent an emerging third intervention modality for patients with established or high-risk muscle wasting. Anamorelin, a ghrelin receptor agonist approved in Japan for cancer cachexia, stimulates appetite and promotes anabolic signaling with demonstrated efficacy in lean body mass preservation. Selective androgen receptor modulators such as enobosarm have shown muscle-anabolic effects in cancer patients with fewer androgenic side effects than testosterone. Myostatin inhibitors and activin receptor type IIB antagonists—including experimental agents such as bimagrumab—directly target the myostatin/FoxO3 axis identified as a central driver of therapy-induced muscle wasting in this review, and represent a mechanistically rational pharmacological approach deserving of clinical validation in immunotherapy combination settings. Anti-inflammatory agents including JAK inhibitors may also mitigate STAT3-mediated muscle catabolism, though their potential interference with immunotherapy efficacy requires careful evaluation (147).

The timing and coordination of these protective interventions relative to immunotherapy cycles requires prospective investigation. A pre-habilitation approach—initiating resistance exercise and nutritional optimization two to four weeks before combination immunotherapy commencement—may establish sufficient anabolic reserve to buffer the catabolic insult of early treatment cycles. Integrating muscle monitoring biomarkers (CK, myostatin, EIM phase angle) with intervention thresholds would enable precision-guided dose escalation of protective strategies, analogous to dose-modification algorithms already established for hematological toxicity. Future research should also examine potential interactions between muscle-protective interventions and immunotherapy efficacy, given emerging evidence that skeletal muscle-derived myokines including irisin and IL-6 exert immune-modulatory effects within the TME (46).

### 5.2 Future mechanistic and translational research priorities

In recent years, substantial advancements have been achieved in interdisciplinary research on tumor immunotherapy, TME, and skeletal muscle biomechanics. Within the domain of tumor immunotherapy, researchers have elucidated the mechanisms by which the immune system identifies and targets tumor cells, resulting in the development of innovative therapies such as ICIs, CAR-T cell therapy, and tumor vaccines. These therapies have significantly enhanced the prognosis of specific cancer types (133). The TME is a critical factor influencing tumorigenesis, progression, and therapeutic outcomes. Evidence suggests that dynamic alterations within the TME can significantly affect the efficacy of immunotherapy, as the accumulation of immunosuppressive cell populations or aberrant tumor vasculature may diminish therapeutic effectiveness (148). The study of skeletal muscle biomechanics is increasingly intersecting with oncology research, with investigators examining how biomechanical properties including muscle tension and shear force affect tumor growth, infiltration, and metastasis. Research has indicated that muscle function and biomechanical status of cancer patients are critical indicators of quality of life, potentially influencing treatment tolerance and efficacy (149).

Despite these advances, several mechanistic gaps remain that are distinct from those identified in our prior review (4) and represent the most pressing priorities for the next phase of research. First, the relative contributions of each of the five combination regimens to therapy-specific versus shared TME-muscle injury pathways require prospective delineation in patient-matched biobank studies correlating circulating cytokine profiles, muscle biopsy histology, and biomechanical endpoints. Second, the fiber-type-specific regulation of MuRF1 and Atrogin-1 expression under different cytokine environments—particularly the differential responses of type I and type II fibers to myostatin/activin versus IL-6/STAT3 signaling—remains poorly characterized and represents a tractable research question with direct clinical relevance for designing fiber-targeted interventions. Third, the bidirectional relationship between satellite cell senescence induced by chronic TGF-β exposure and the accumulation of senescence-associated secretory phenotype (SASP) factors within the TME warrants investigation as a self-amplifying loop driving both muscle regenerative failure and tumor immune evasion. Fourth, the interaction between corticosteroid-induced myopathy and immune myositis in patients receiving irAE management requires dedicated mechanistic study to establish differential diagnostic criteria and optimized tapering protocols that minimize steroid myopathy risk while achieving irAE control. Fifth, the integration of artificial intelligence-driven multimodal data analysis—combining imaging-derived muscle phenotypes, transcriptomic profiling of circulating cell-free RNA, and wearable sensor-derived functional data—holds promise for developing predictive algorithms that identify patients at high risk of severe muscle dysfunction before clinical manifestation, enabling pre-emptive intervention. Sixth, sex-stratified and age-stratified clinical studies are needed to validate the population-specific cut-offs for muscle assessment and intervention thresholds proposed in this review, converting the mechanistic heterogeneity described in Section 3.6 into actionable clinical decision rules (150).

The integration of traditional and emerging assessment techniques offers comprehensive tools for analyzing the interplay between tumor immunotherapy, tumor microenvironment, and skeletal muscle biomechanics (151). Tackling these challenges requires a multidisciplinary approach, further refinement of technical methodologies, and the development of cross-disciplinary evaluation frameworks.

In summary, the future research agenda emerging from this review is distinguished from prior work by its emphasis on intervention translation, population stratification, and the clinical operationalization of mechanistic insights—moving the field from descriptive characterization of immunotherapy-related muscle injury toward actionable, precision strategies that preserve skeletal muscle function without compromising antitumor efficacy.

## 6 Conclusion

This review provides a comprehensive and structurally integrated analysis of the mechanisms, clinical confounders, assessment methods, and protective strategies relevant to skeletal muscle dysfunction in the context of combination immunotherapy. Extending beyond prior work from our group that focused primarily on single-agent ICI-induced myositis (4), this review systematically maps the heterogeneous muscle toxicity profiles of five distinct combination regimens—chemotherapy, targeted therapy, gene therapy, tumor vaccines, and CAR-T cell therapy—against a unified framework of shared TME-muscle crosstalk pathways and therapy-specific injury mechanisms.

The shared mechanistic foundation across all combination regimens encompasses the CAF-TGF-β/SMAD3 fibrotic axis, Warburg-driven lactate-mediated metabolic suppression, MDSC/Treg arginine depletion, and the interconnected IL-6/TNF-α inflammatory cascade converging on the myostatin/FoxO3/MuRF1/Atrogin-1 atrophy circuit and defective mitophagy. Superimposed upon this shared basis, each modality carries distinct myotoxic signatures: cisplatin-driven ROS accumulation and indirect cholinergic oxidative injury, bevacizumab-mediated ischemic metabolic stress, gene vector immunogenicity, adaptive immune-driven transient vaccine myalgia, and CRS-mediated systemic metabolic storm in CAR-T therapy.

This review introduces three dimensions absent from prior analyses. First, it delineates the differential vulnerability of fast-twitch glycolytic versus slow-twitch oxidative fibers and the mechanism by which sustained cytokine exposure drives satellite cell senescence, converting reversible acute injury into chronic progressive wasting. Second, it systematically addresses two major clinical confounders—corticosteroid use and patient demographic heterogeneity (age, sex, hormonal status)—that critically modulate individual muscle injury severity and recovery potential, underscoring the need for population-stratified monitoring and intervention protocols. Third, it consolidates evidence-based muscle-protective strategies—resistance exercise, leucine-enriched nutritional supplementation, anti-cachexia pharmacotherapy, and pre-habilitation approaches—within a framework that integrates intervention timing with immunotherapy cycle schedules.

The assessment framework described in this review—spanning handheld dynamometry, DXA, CT, isokinetic testing, ultrasound elastography, and EIM—provides a tiered, multimodal toolkit with defined sensitivity profiles for early versus established muscle injury detection. The proposed integration of these tools into prospective clinical trial design, including composite endpoints, pre-specified stratification, and tiered monitoring algorithms, offers a translational pathway toward regulatory recognition of muscle function as a meaningful co-primary endpoint in immunotherapy trials.

Collectively, the mechanistic, clinical, and interventional framework developed in this review supports a paradigm shift in oncological care: from an exclusive focus on tumor eradication to a holistic approach that actively preserves skeletal muscle function as an integral component of treatment planning, toxicity management, and survivorship strategy. Realizing this paradigm requires coordinated interdisciplinary collaboration among oncologists, immunologists, sports medicine specialists, physiotherapists, and nutritionists, guided by the precision stratification principles outlined throughout this work. As combination immunotherapy continues to evolve and expand across tumor types, systematic incorporation of skeletal muscle health monitoring and protection into standard clinical protocols will be essential to sustain patient functional capacity, treatment adherence, and long-term quality of life.
